# Reactive Laser Additive Manufacturing of Hierarchically Structured Aerogels

**DOI:** 10.1002/adma.73352

**Published:** 2026-05-08

**Authors:** Shuichiro Hayashi, Ankit Das, Marco Rupp, Elizabeth Stump, Joshua Miller, Michele L. Sarazen, Craig B. Arnold

**Affiliations:** ^1^ Princeton Materials Institute Princeton University Princeton New Jersey USA; ^2^ Department of Mechanical and Aerospace Engineering Princeton University Princeton New Jersey USA; ^3^ Department of Chemical and Biological Engineering Princeton University Princeton New Jersey USA

**Keywords:** additive manufacturing, energy storage, graphitic aerogels, laser materials processing, vapor‐liquid‐solid growth

## Abstract

As demands for sustainable and scalable energy materials manufacturing accelerate, additive manufacturing (AM) remains largely limited to passive shaping of predefined precursors. Here, we introduce reactive laser AM, in which precursor composition is designed to transform the printing step itself into a chemically active stage of materials synthesis. Incorporating eutectic alkali halide salts into protein‐based powders converts localized laser heating into transient reaction environments that drive vapor‐phase chemistry, surface etching, and in situ hierarchical growth without external reagents or solvents. This internally activated reactivity enables the rapid formation of graphitic aerogel monoliths with multilevel architecture—macroporous frameworks decorated with microtubular arrays and nanoscale features—within seconds in a single process. As energy storage electrodes, these hierarchically structured aerogels exhibit a tenfold enhancement in gravimetric capacitance (∼162 F g^−1^) relative to salt‐free counterparts. By engineering reactivity through feedstock design, this work reframes laser AM as a dynamic platform for reaction‐driven materials‐by‐design.

## Graphical abstract

Reactive laser additive manufacturing transforms printing into a chemically active synthesis step. Salt‐enabled transient reaction environments drive in situ formation of hierarchically structured graphitic aerogels with microtubular and nanoscale features in seconds. This internally programmed reactivity yields over tenfold capacitance enhancement, establishing a scalable solvent‐free pathway for reaction‐driven materials‐by‐design.

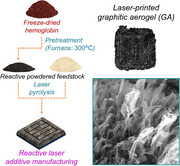

## 1 Introduction

With the global transition to electrification, advancing portable and structural energy devices requires electrodes that are simultaneously lightweight, high‐performance, and scalable; all challenges that conventional carbon‐based thin films struggle to meet [1]. Graphitic aerogels (GAs), or three‐dimensionally macroporous assemblies of carbon nanomaterials, have emerged as promising alternatives. Their ultralow density, high surface area, and interconnected conductive networks enable efficient mass and charge transport while maximizing utilization of 3D volume, making them particularly attractive for electrochemical systems where multifunctionality must be balanced against stringent mass constraints [2, 3, 4, 5, 6]. Moreover, increasing attention has been directed toward deriving such architectures from renewable or biomass‐based precursors, providing a pathway toward more sustainable and scalable materials manufacturing [7, 8].

Additive manufacturing (AM) offers a powerful route to tailor GA architectures with digital precision. By enabling control over geometry and shape, AM provides design flexibility beyond traditional bulk synthesis, allowing custom‐shaped devices precisely matched to application geometries for compact and lightweight systems [9, 10, 11]. Among existing techniques, ink‐based extrusion printing of graphene oxide (GO) and related carbon inks dominates. While effective, this approach relies on complex formulations, costly binders, and sacrificial additives, and requires subsequent chemical reduction or thermal annealing to achieve monolithic macroporous GAs with sufficient conductivity [1]. These steps are time‐intensive, tedious, and often environmentally hazardous, limiting scalability. Moreover, structural parameters such as multiscale porosity, surface area, and hierarchical architecture are largely predetermined by the precursor ink and template chemistry, making it extremely challenging to introduce additional multilevel features without complete reformulation of the printing protocol. Hence, more dynamic AM frameworks that offer lower‐cost material options and shift from conventional static AM approaches, where the printed structure merely mirrors the properties of its precursor, are critical to realizing true digital manufacturing of advanced energy devices.

Laser‐based powder bed fusion (PBF), a technique widely used for metal AM, has recently been adapted to print GAs via laser pyrolysis of protein‐based precursors [12, 13]. In this process, hemoglobin powder is locally converted into graphitic frameworks using a continuous‐wave (CW) laser, simultaneously achieving carbon synthesis and shape definition in a single dry step. By eliminating inks, binders, and corrosive etchants, and by using biogenic feedstocks such as animal blood, this method offers a potentially scalable and sustainable route. Importantly, digital control of laser parameters enables concurrent tuning of material chemistry and geometry. Despite these advances, laser‐printed GAs typically lack hierarchical porosity and surface features, resulting in smooth surfaces. While advantageous for electrical and thermal transport [13], this limits performance in energy storage, where interfacial reactions and charge transport scale with accessible surface area. Therefore, a strategy to introduce hierarchical porosity in situ during laser printing, without compromising printability or resolution, would represent a critical leap toward adaptive laser manufacturing of multifunctional GAs.

Here, we introduce a reactive laser printing strategy in which the chemical activity of the printing process is engineered through precursor design rather than external reagents or post‐processing. By incorporating eutectic alkali halide salts into protein‐based powders, localized laser heating triggers transient reaction environments that activate in situ chemical transformations concurrent with aerogel formation. In this framework, the laser does not merely shape a predefined material, but dynamically mediates chemical reactions that restructure the carbon network as it is printed. The resulting GAs exhibit enhanced surface complexity and functionality compared to salt‐free analogues, leading to improved electrochemical behavior. More broadly, this approach establishes reactive AM as a route to architected carbon materials whose structure and function emerge from internally driven reactions during fabrication.

## 2 Results

### 2.1 Digital Printing of Macroporous Frameworks

**FIGURE 1:**
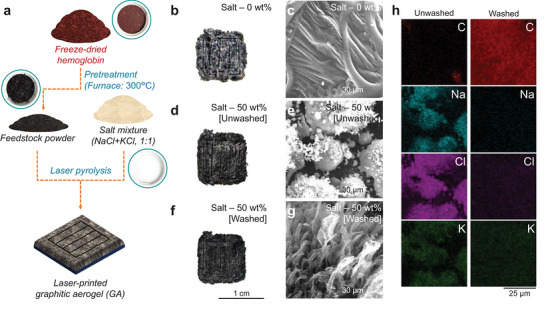
Salt‐assisted laser printing of graphitic aerogels with enhanced surface morphology. (a) Schematic illustration of the laser printing process. Insets are photographs of the powdered feedstocks. Photograph and SEM images of the aerogels printed (b,c) without salt (0 wt.%), (d,e) with 50 wt.% salt before water washing, and (f,g) with 50 wt.% salt after water washing. (h) Elemental mapping images of the unwashed surface in (e) and washed surface in (g).

GAs were fabricated via a PBF‐inspired process, schematically illustrated in Figure 1a. Pristine hemoglobin powder was first pretreated at 300°C to induce partial aromatization and enhance carbon yield, mitigating rapid volatilization and structural collapse during subsequent laser processing [13]. The resulting powder was then spread as a bed and selectively irradiated using a CW infrared laser beam under an inert atmosphere. The spatial confinement of heat within the digitally defined laser path enables concurrent material synthesis and geometric control, effectively “printing” freeform GAs through coupled precursor fusion and graphitic conversion.

Upon laser irradiation, the protein powders rapidly soften and coalesce into a transient viscous phase, enabling interparticle fusion into a continuous monolithic structure. As pyrolysis progresses, volatile release generates bubbles within this viscous medium, driving a self‐foaming process that expands the material while it simultaneously undergoes carbonization and solidification, ultimately yielding a porous GA framework [14]. By raster scanning the pretreated powder bed in a square design, ultralight yet mechanically robust graphitic monoliths can be directly printed, which are sufficiently stiff to be readily handled without noticeable deformation (Figure 1b) [13]. Using this approach, salt‐free prints produced centimeter‐scale GAs within seconds (<30 s) under N_2_, with a smooth macroporous framework (pore diameter: ∼100–300 µm; Figure 1b,c and Figure S1) and an ultralow volumetric density (∼0.012 ± 0.002 g cm^−3^; Figure S2).

### 2.2 Salt‐Assisted Growth of Microtubular Surface Structures

To induce microscale surface features during printing, a eutectic NaCl/KCl mixture (1:1 molar ratio) was incorporated into the feedstock at 50 wt.% of the total powder mass. Despite the high additive loading, the printed monoliths retained their precise geometric architecture (Figure 1d,e). After water washing, the monoliths exhibited no structural distortion (Figure 1f,g) and only a minor change in volumetric density (∼0.018 ± 0.003 g cm^−3^; Figure S2). Prior to washing, scanning electron microscopy (SEM) revealed aggregated salt crystallites coating the surface (Figure 1e,h). After salt removal (Figure S3), the underlying aerogel framework was densely covered by carbon‐rich tubular microstructures firmly anchored to its surface (Figure 1g,h), yielding a coral‐like topology. Neither NaCl nor KCl alone produced comparable features (Figure S4), indicating a synergistic melting behavior during printing. The use of common alkali halide salts thus enables environmentally benign microporous structuring without toxic etchants, while allowing salt recovery and reuse through simple water dissolution.

**FIGURE 2:**
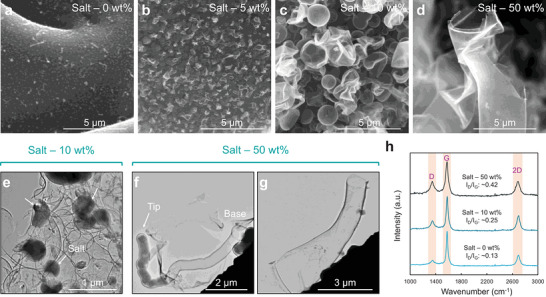
Surface evolution of laser‐printed graphitic aerogels with increasing salt concentration. SEM images of aerogels printed with (a) 0, (b) 5, (c) 10, and (d) 50 wt.% salt. TEM images of surface features formed at (e) 10 wt.% and (f,g) 50 wt.% salt; (f) shows a closed‐tip graphitic microtube, whereas (g) shows an open‐tip microtube. (h) Raman spectra of aerogels printed with different salt concentrations. All salt‐containing samples were water‐washed prior to analysis.

GAs were printed with increasing salt concentrations to systematically probe morphological evolution (Figure 2a–d). Without salt, the GA surface remained smooth and featureless (Figure 2a). At 5 wt.%, blister‐like nanostructures emerged (∼450 ± 100 nm; Figure 2b), while 10 wt.% yielded larger spherical features, some partially collapsed (∼1.4 ± 0.61 µm; Figure 2c). At 50 wt.%, high‐aspect‐ratio hollow microtubes populated the surface (∼1.7 ± 1.33 µm; Figure 2d). Bright‐contrast regions embedded within these structures indicated local compositional heterogeneity, with energy dispersive X‐ray (EDX) spectroscopy confirming partial salt entrapment during growth (Figure S5) [15]. This was especially evident in closed‐tip tubes, where salt was frequently localized at the nib‐like apex (Figure S6). Potassium signals were visibly weaker than sodium, due to its higher volatility resulting from a lower boiling point and weaker metallic bonding [16, 17].

Transmission electron microscopy (TEM) offered deeper insight into the evolution of graphitic architecture. In salt‐free GAs, well‐aligned graphitic layers dominated the structure, forming flat basal planes (Figure S7). With 10 wt.% salt, circular features with dark edge contrast indicated the presence of hollow spherical shells (Figure 2e), often containing residual salt crystallites. High‐resolution TEM revealed that the shell walls were ∼10 nm thick and consisted of multilayered graphitic carbon (Figure S8). At 50 wt.%, well‐defined tubular structures became prominent, with residual salt often trapped near the closed tips (Figure 2f, white arrow; Figure S9). Their wall thickness increased to ∼35 nm, while open‐tipped tubes exhibited slightly thinner walls (∼25 nm; Figure 2g and Figure S10).

Raman spectroscopy for all samples shows a weak D band (∼1350 cm^−1^) and prominent G (∼1580 cm^−1^) and 2D (∼2700 cm^−1^) bands, indicating sp^2^‐rich graphitic carbon formation (Figure 2h). The D‐to‐G band intensity ratio (I_D_/I_G_) increases from ∼0.13 (0 wt.%) to ∼0.42 (50 wt.%), reflecting increased defect density with salt incorporation. Further analysis of the 2D band reveals that the 50 wt.% sample exhibits a downshift in the 2D peak position to 2688 cm^−1^ compared to 2696 cm^−1^ for the 0 wt.% sample (Figure S11), consistent with increased curvature‐induced strain in tubular structures [18]. In addition, the full width at half maximum (FWHM) of the 2D band increases from 58.6 cm^−1^ (0 wt.%) to 79.8 cm^−1^ (50 wt.%), indicating reduced out‐of‐plane structural coherence with increasing salt content. This suggests that while graphitic ordering is preserved, salt‐assisted processing introduces curvature and structural disorder at smaller length scales.

These observations indicate that the bulk GA framework forms primarily through laser pyrolysis of the protein precursor, whereas the surface microtubes arise secondarily via a salt‐assisted vapor–liquid–solid (VLS)‐like mechanism during printing [19, 20, 21]. In this mechanism, laser‐induced heating establishes a transient multiphase environment resembling that required for classical VLS growth: the GA surface acts as the solid substrate, molten alkali halide droplets as the liquid template, and carbon‐rich vapors from laser pyrolysis as the vapor precursor. The eutectic mixture melts at temperatures that overlap with the graphitization regime [15], facilitating templated graphitic growth at the droplet–aerogel interface. Smaller coalesced droplets at lower salt loadings yield nanoscale blisters (Figure 2b), whereas larger droplets form microspheres (Figure 2c). Continued precipitation and asymmetric deposition at the liquid–solid boundary drive anisotropic growth, ultimately forming high‐aspect‐ratio hollow microtubes (discussed in Supporting Information; Figures S12–S17) [22]. These microtubular surface features were absent in furnace‐pyrolyzed GAs prepared from the same salt‐containing hemoglobin feedstock, indicating that rapid laser heating is essential for their formation (Figure S18).

### 2.3 Vapor‐Phase Reactions for Submicron Features

**FIGURE 3:**
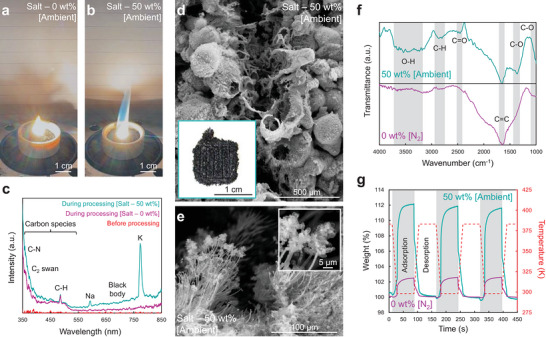
Reactive laser printing of hierarchical graphitic aerogels under ambient conditions. Photographs of the laser‐printing process in ambient with (a) 0 and (b) 50 wt.% salt. (c) Optical emission spectra measured before and during laser processing in ambient. (d) Surface SEM image of the aerogel printed with 50 wt.% salt; inset shows a photograph of the printed monolith. (e) SEM image showing nanoparticles adhered to tubular surface features; inset shows a higher‐magnification view of the tips. (f) FTIR spectra obtained from salt‐free GAs printed in N_2_ (pink) and salt‐assisted GAs printed in ambient conditions (blue). (g) Thermogravimetric CO_2_ adsorption–desorption analyses for salt‐free (0 wt.% salt in ambient) and salt‐assisted (50 wt.% salt in ambient) GAs. The thermogram is indicated in red.

To exploit the additional reactive interfaces introduced by the salts, laser processing was conducted under ambient conditions. Pristine protein powder processed in air reacted violently with environmental gases, releasing dense dark vapors (Figure S19a) and causing substantial mass loss (Figure S20). In contrast, pretreated protein powders exhibited reduced vapor evolution (Figure 3a) and lower mass loss. Bright light emission was observed at the focal volume (Figure 3a), in sharp contrast to the dim glow seen for pristine powders (Figure S19a). These differences are attributed to the formation of aromatic domains during pretreatment, which serve as thermal stabilizers [13]. This enhanced thermal stability allows higher localized temperatures, consistent with the stronger black‐body radiation observed as bright emission.

Incorporation of the eutectic salt mixture into the pretreated powder further intensified vapor evolution, producing a stable blue‐hued stream from the focal volume (Figure 3b). Although pristine protein powders mixed with salt also showed increased vapor evolution (Figure S19b), a similar stable blue‐hued stream was not confirmed. Optical emission spectroscopy measurements before and during laser processing revealed emissions from carbon species, such as C─N, C_2_, and C─H, regardless of salt addition (Figure 3c), consistent with volatile evolution during high‐temperature degradation of organic and graphitic matter [23, 24]. An increase in the spectral baseline further indicated elevated black‐body radiation. Additional sharp emission peaks corresponding to sodium and potassium appeared only for salt‐containing precursors, indicating that the visible blue vapor originates from alkali halide evaporation at temperatures exceeding 1400°C. The stronger potassium emission relative to sodium aligns with weaker surface EDS signals observed earlier (Figure S6), consistent with the higher volatility of potassium salts. The enhanced black‐body radiation likely arises from a salt‐matrix effect that increases plasma temperature and electron excitation, leading to stronger emissions [25]. Despite enhanced vapor release, total mass loss decreased by over 10% in the presence of salts (Figure S20). This suggests that the blue‐hued visible vapor consisted primarily of alkali species, whereas salt‐free processing released largely transparent hydrocarbon vapors. Furthermore, transient molten salt droplets likely improved local wettability and promoted redeposition of carbonaceous vapors [26], contributing to the observed mass retention.

Monolithic GAs with well‐defined geometries were printed under ambient conditions at a salt loading of 50 wt.% (Figure 3d, inset). However, the underlying framework morphology shifted from the sheet‐like structure observed in N_2_ (Figure S21) to a more cellular bubble‐like architecture with comparable pore diameters (Figure 3d and Figure S22). Tubular features also formed both externally and internally in air (Figure S23), though they appeared more irregular, with wrinkled walls and complex orientations. Additionally, nanoparticle aggregates were observed along tube peripheries and tips even after water washing (∼868 ± 332 nm, Figure 3e and Figure S24). These likely correspond to soot‐like carbon‐black particulates that adhered to the surface during laser processing in air. Raman spectroscopy confirmed sp^2^‐rich carbon formation under ambient conditions (Figure S25), though the I_D_/I_G_ ratio increased from ∼0.42 (N_2_) to ∼0.92, indicating greater structural disorder from mild oxidation. The significantly higher atmospheric CO_2_ peak at ∼2330 cm^−1^ may result from reduced overall Raman intensity due to increased surface roughness.

Beyond morphological changes, the chemical and nanoscale structure of the aerogels also differed between salt‐free and salt‐assisted conditions. Fourier‐transform infrared (FTIR) spectroscopy of salt‐free GAs (0 wt.% in N_2_) showed a single dominant C═C stretch, consistent with sp^2^‐rich carbon observed by Raman. In contrast, salt‐assisted GAs printed in ambient exhibited additional C─O, C═O, and O─H signatures (Figure 3f), indicating incorporation of oxygen‐containing functional groups during reactive processing. To probe differences in ultrafine porosity, CO_2_ adsorption–desorption measurements were performed (Figure 3g). Both samples showed reversible CO_2_ uptake; however, the salt‐assisted GA exhibited a sixfold higher adsorption capacity, reflecting the combined effects of enhanced sub‐nanometer porosity and increased surface functionality induced by vapor‐phase reactions. These distinctions were not resolved by conventional N_2_ physisorption (Figure S26), consistent with prior reports that N_2_ at 77 K underestimates accessible surface area in oxygen‐rich carbon monoliths due to slow diffusion and restricted access to ultrafine pores [27, 28]. In contrast, CO_2_, with its higher kinetic energy and stronger quadrupole interactions, more effectively probes partially blocked, functionalized, or sub‐nanometer pores, revealing differences otherwise inaccessible by N_2_‐based analysis.

These changes arise from synergistic effects between the salts and the ambient atmosphere under the extreme non‐equilibrium conditions of laser processing. Released salt vapors, particularly potassium species as indicated by spectroscopy (Figure 3c), can catalytically react with H_2_O, O_2_, and CO_2_ to initiate surface etching and nanoparticle redeposition through cyclic reactions (discussed in Supporting Information) [29, 30]. Under inert N_2_ conditions, even with salt addition, these reactions are self‐limited by the heteroatom content of the protein‐based precursor, resulting in a more controlled and static structural evolution. In contrast, the synergistic coupling between salt vapors and ambient reactive species drives dynamic etching, carbon redeposition, and hierarchical structure formation, yielding centimeter‐scale monolithic aerogels that integrate macroporous frameworks, tubular features, nanoparticulate decorations, and nanofeatures without compromising printability or resolution. Such reactions are unique to laser printing; under conventional pyrolysis, processing in air leads to structural disintegration and no residual material (Figure S27).

The structural features and their corresponding formation mechanisms are summarized in Table 1 (Figure S28). On this basis, the hierarchical structure of the GAs can be tuned across multiple length scales through processing conditions. At the macroscale, the overall monolith geometry and dimensions are governed by laser energy delivery and thermal coupling, which define the spatial extent of precursor fusion and graphitization. At the microscale, the macroporous framework arises from bubble nucleation and growth within the transient viscous phase and is modulated by local thermal gradients that control gas evolution and foaming dynamics. Accordingly, such structural features can be controlled through laser processing parameters such as wavelength, intensity distribution, and resident time, which determine local energy input and thermal diffusion during printing.

**TABLE 1:** Hierarchical structural features, characteristic size scales, and formation mechanisms.

Structural feature	Characteristic size	Formation mechanism
Monolith	>>1 mm	Laser‐induced precursor fusion and pyrolysis
Macropores	∼10–500 µm	Bubble nucleation and growth during pyrolysis
Tubular structures	∼500 nm–10 µm	VLS‐like growth on molten salt droplets
Particles	∼100 nm–1 µm	Gas‐phase precipitation of vapor species
Nanopores	<<1 nm–20 nm	Chemical etching by reactive species

At smaller length scales, the emergence of graphitic tubular structures is consistent with a VLS‐like growth mechanism (Figure S15), in which carbon vapor deposits onto transient molten salt droplets; accordingly, droplet characteristics and reaction duration influence their formation. In addition, nanoparticulate and nanoporous features arise from gas‐phase reactions between volatilized species and the surrounding atmosphere, as well as concurrent chemical etching processes. These features can therefore be adjusted through the choice of reactive environment and feedstock chemistry. These parameters provide multiple handles for tailoring structure across length scales, although they currently remain inherently coupled under the present processing conditions.

### 2.4 Electrochemical Performance of Hierarchical Structures

**FIGURE 4:**
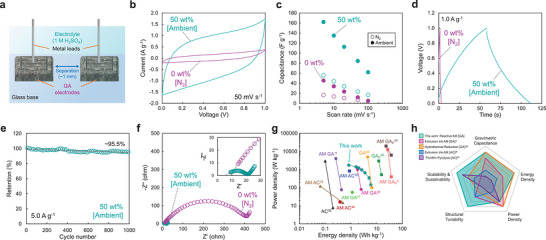
Electrochemical performance of laser‐printed graphitic aerogels in symmetric supercapacitors. (a) Schematic of the fabricated symmetric GA supercapacitor. (b) CV curves at 50 mV s^−1^ for salt‐free GAs printed in N_2_ (pink) and salt‐assisted GAs printed in ambient conditions (blue). (c) Gravimetric capacitance as a function of scan rate for salt‐free (pink) and salt‐assisted (blue) GAs, printed in N_2_ (open circles) or ambient (closed circles). (d) CP curves at 1.0 A g^−1^ for salt‐free GAs printed in N_2_ (pink) and salt‐assisted GAs printed in ambient (blue). (e) Cycling stability of salt‐assisted GAs printed in ambient at 5.0 A g^−^^1^. (f) Nyquist plots for salt‐free (pink) and salt‐assisted (blue) GAs. Inset is the expanded high‐frequency region showing the salt‐assisted GA plot. (g) Ragone plot benchmarking salt‐assisted GA electrodes against representative carbon‐based supercapacitors. (h) Radar plot of carbon‐based electrode fabrication techniques.

Laser‐printed GAs were evaluated as electrodes in symmetric planar supercapacitors (Figure 4a). Cyclic voltammetry (CV) curves of both salt‐free and salt‐assisted GAs exhibited rectangular profiles (Figure 4b), indicative of electric double‐layer capacitive behavior. Gravimetric capacitances extracted from CVs at various scan rates (Figures S29 and S30) are summarized in Figure 4c. Values were normalized with respect to the combined mass of the two GA electrodes. At 5 mV s^−1^, the salt‐assisted GAs printed in ambient achieved ∼162.2 F g^−1^, significantly higher than those printed in N_2_ (∼56.0 F g^−1^), and far exceeding salt‐free counterparts printed in ambient (∼45.6 F g^−1^) or N_2_ (∼16.8 F g^−1^). The hierarchically structured salt‐assisted GA (50 wt.% in ambient) showed over a ten‐fold enhancement relative to the non‐structured salt‐free GA (0 wt.% in N_2_). GAs printed in ambient conditions consistently exhibited higher capacitances than those printed in N_2._ This enhancement is consistent with the increased sub‐nanometer porosity and surface functionality identified by CO_2_ adsorption (Figure 3g), which together improve ion‐accessible surface area and charge storage [31]. Chronopotentiometry (CP) further confirmed the enhanced capacitive behavior of the laser‐printed electrodes (Figure 4d and Figure S31). Both salt‐free (0 wt.% in N_2_) and salt‐assisted (50 wt.% in ambient) electrodes displayed triangular charge–discharge curves; however, the salt‐assisted GAs showed longer discharge durations and smaller voltage drops, consistent with improved charge storage performance. Notably, the salt‐assisted GAs demonstrated excellent cycling stability, retaining nearly full capacitance after 1000 charge–discharge cycles at a high current density of 5 A g^−1^ (Figure 4e), comparable to other carbon‐based supercapacitors [1]. While trace amounts of residual salt remain within the GAs after washing, their influence on electrochemical performance is considered negligible under the present conditions. No additional redox features are observed in the CV or CP profiles (Figure 4b,d), and the electrodes exhibit stable cycling behavior without accelerated degradation. Given the substantially larger mass of the electrolyte relative to the electrode, any leached ionic species are expected to be highly diluted, minimizing their impact on overall device performance.

Electrochemical impedance spectroscopy (EIS) revealed that the salt‐assisted GAs possessed a slightly smaller equivalent series resistance of ∼5.45 Ω than the salt‐free counterparts of ∼8.96 Ω (Figure 4f). As both devices share identical configurations, this minor reduction may reflect improved electrode–electrolyte contact, consistent with the increased abundance of oxygen‐containing surface groups that enhance wettability (Figure 3f). More notably, the Nyquist plot of the salt‐assisted GA showed a semicircle nearly an order of magnitude smaller than that of the salt‐free counterpart (∼10 Ω vs. ∼400 Ω), indicating a greatly reduced charge‐transfer resistance (Figure 4f; inset). The hierarchically porous structure, together with enhanced surface functionality, of the salt‐assisted GA provide greater accessible surface area and more efficient ion‐transport pathways, compensating for its lower graphitic crystallinity. In the low‐frequency region, both electrodes displayed sloped tails characteristic of finite‐length diffusion; however, the salt‐assisted GA showed a slightly steeper slope (Figure S32), indicating behavior closer to an ideal capacitive response with reduced diffusion limitation. These results align with the enhanced rate performance observed in CP measurements and confirm that the hierarchical porous network substantially improves ion accessibility and charge‐storage kinetics.

Energy and power densities extracted from CP data are summarized in the Ragone plot (Figure 4g). Salt‐assisted GAs printed in ambient conditions achieved energy densities of ∼0.83–5.97 Wh kg^−1^ across power densities of ∼145–1752 W kg^−1^, outperforming many state‐of‐the‐art activated carbon (AC)‐based devices (Figure 4g, triangles), including both bulk [32] and AM [33, 34, 35] electrodes. Their performance is also comparable to, and in some cases exceeds, that of recently reported bulk [36] and AM [11, 37, 38] GA electrodes (Figure 4g, circles). Devices that outperform the present material are primarily pseudocapacitive GAs (GA_P_) [9, 39, 40] where embedded redox‐active species enhance charge storage (Figure 4g, squares). Therefore, among purely carbon‐based electrodes, the present hierarchically structured GA ranks among the highest‐performing. Notably, this performance is achieved using biogenic‐waste‐derived precursors, indicating the competitiveness of the present system relative to both conventional and biomass‐derived carbon electrodes.

The laser‐based reactive AM process uniquely integrates high electrochemical performance with exceptional manufacturing adaptability (Figure 4h and Table S1). Self‐standing thin‐film AC electrodes, while industrially scalable, offer minimal control over architecture and limited capacitance. Transitioning to three‐dimensionally macroporous GAs improves electrochemical performance but sacrifices scalability and sustainability, as synthesis involves toxic etchants and multistep treatments [41]. AM introduces new levels of structural tunability, enabling tailored properties and conformal geometries for device integration; however, it often sacrifices electrochemical performance due to the need for binders and sacrificial templates for ink‐based printing. In contrast, the present approach eliminates these constraints and is compatible with sustainable low‐cost biogenic feedstocks and common salts to achieve digitally controlled structure and chemistry in a single rapid dry step. This bridges long‐standing trade‐offs among hierarchical tunability, sustainability, and throughput while delivering energy storage performance competitive with state‐of‐the‐art systems. Remaining challenges primarily concern power performance, as millimeter‐scale electrode thickness limits ion diffusion and charge transport. Incorporating controlled lattice geometries or thinner architectures may further enhance volumetric utilization and optimize the energy–power balance [42, 43].

## 3 Conclusions

Harnessing feedstock composition, we realize hierarchically porous functional GAs through laser‐triggered reactive chemical processes during AM. Incorporating eutectic alkali halide salts into protein‐based precursors enables localized laser irradiation to simultaneously form macroporous frameworks and drive transient VLS‐like growth of microscale tubules that decorate the bulk. Processing under ambient conditions further activates cyclic surface reactions that produce nanoscale particulates, etched pores, and active functional groups, achieving GAs with multilevel hierarchical complexity. This internally driven structuring directly enhances material performance, where the resulting aerogels deliver over a tenfold increase in gravimetric capacitance relative to salt‐free counterparts, placing them among the highest‐performing purely carbon‐based supercapacitors reported to date.

Beyond performance, this approach redefines AM by remodeling the printing step into a reactive stage of materials synthesis, rather than a passive assembly of precursors. Transient thermal, chemical, and environmental conditions actively shape composition, morphology, and surface functionality within a single, rapid, and solvent‐free step. This coupling transforms parameters such as ambient gases or volatized species into active inputs for tuning material chemistry and architecture in real time, unveiling new links between process, environment, structure, and property; factors that are central to next‐generation manufacturing. These capabilities position reactive laser printing as a scalable and adaptable route for architected material fabrication, advancing a broader vision of laser‐driven reaction‐based materials‐by‐design for energy and catalysis applications.

## 4 Methods

### 4.1 Feedstock Preparation

Freeze‐dried bovine hemoglobin (MP Biomedical, USA) was used as the precursor. Unless specified as pristine powder, such as in Figure S19, the hemoglobin was heat‐treated in a tube furnace under continuous N_2_ flow to prepare the pretreated powder feedstock. The temperature was ramped to 300°C at a heating rate of 35°C min^−1^, held isothermally for ∼2 h, and then allowed to cool naturally to ambient temperature. The resulting solid was ground into a fine powder and mixed with a 1:1 molar ratio salt mixture of NaCl and KCl at various weight percentages. For example, to prepare a 50 wt.% composite feedstock, equal masses of the pre‐treated protein‐based powder and the salt mixture were combined.

### 4.2 Laser Experiments

The powdered feedstock was prepared as a ∼1 cm‐thick bed in a holder and placed inside a windowed flow chamber equipped with a gas inlet and a vacuum outlet (Figure S33). A CW laser (YLR‐100, IPG Photonics, USA) with a central wavelength of ∼1070 nm was focused and raster‐scanned across the powder surface using a digital galvanometer scanner (Focus Shifter, Raylase GmbH, Germany) [13]. The laser spot diameter at the surface was ∼500 µm. Unless otherwise noted, the laser power and scanning speed were fixed at 10 W and 2 mm s^−1^, respectively. These parameters were selected based on prior studies demonstrating that this regime yields structurally continuous and electrically conductive laser‐printed GAs [12], while maintaining stable processing conditions [44]. Fixing these conditions enables isolation of feedstock‐dependent effects in the present study. Square‐shaped aerogels were printed using a hatch spacing of 2.00 mm in a single‐layer powder bed. Post‐processing involved rinsing and soaking the printed structures in deionized water for ∼24 h to remove residual salts, followed by drying at ∼80°C for ∼1 h. Any remaining visible debris or loosely adhered carbonized crusts were mechanically removed after drying.

### 4.3 Material Characterizations

SEM images were obtained using an Inspect F50 environmental SEM (Thermo Fisher Scientific, USA). Elemental analyses were performed in conjunction with SEM using an X‐MAX spectroscope (Oxford Instruments, UK). TEM images were obtained using a Talos F200X TEM (Thermo Fisher Scientific, USA). TEM samples were prepared by drop‐casting grounded aerogel powder onto copper‐based grids. Raman spectra were obtained using a laser‐excited LabRAM Aramis spectrometer (Horiba, Japan), with a ∼532 nm excitation wavelength and ∼36 mW laser power. Optical emission spectra were obtained using a fiber‐based CCS200 charge‐coupled device spectrometer (Thorlabs, USA), with a collimator positioned near the laser processing zone for in situ signal collection (Figure S34). FTIR spectra were obtained in reflection mode using a Nicolet iN10 MX spectrometer (Thermo Fisher Scientific, USA).

The volumetric density *D_V_* was calculated according to Equation (1),

(1) $$D_{V}=\frac{m}{w\times l\times h}\;$$

where *m* is the aerogel mass, and *w*, *l*, and *h* are the width, length, and height, respectively, measured using a precision scale and confocal microscope. Mass loss during laser processing (Figure S20) was determined by comparing the total mass of the holder and powder bed before irradiation with that of the holder, residual bed, and printed structure after processing.

CO_2_ adsorption–desorption isotherms were obtained using a TGA 8000 analyzer (PerkinElmer, USA). Desorption cycles were conducted by ramping to ∼383 K under argon at a flow rate of 40 mL min^−1^. Adsorption cycles were conducted by cooling to ∼298 K and flowing 10% CO_2_ in helium for three consecutive cycles at flow rates of 10, 20, and 30 mL min^−1^. Temperature ramps were all conducted at 5 K min^−1^. N_2_ adsorption–desorption isotherms were obtained using a 3Flex physisorption analyzer (Micromeritics, USA) at ∼77 K.

### 4.4 Thermal Simulations

The increase in local temperature induced by CW laser scanning was simulated using a 3D finite element model in COMSOL Multiphysics. The laser heat input *Q* was defined as a spatially varying Gaussian distribution according to Equation (2),

(2) $$Q\;\left(x,y,z\right)=PA\frac{A_{c}}{\pi \sigma_{x}\sigma_{y}}e^{-\left[\frac{{\left(x-x_{0}\right)}^{2}}{2\sigma_{x}^{2}}+\frac{{\left(y-y_{0}\right)}^{2}}{2\sigma_{y}^{2}}\right]}.e^{-A_{c}z}$$

where *P* is the laser power, *A* is the absorptivity, *A_c_* is the absorption coefficient, *σ_x_* and *σ_y_* are the beam radii in the x and y directions, and *x_0_* and *y_0_* are the origin coordinates [45].

During raster scanning, the laser motion was modeled as a linear translation according to Equations (3) and (4),

(3) $$x=x_{0}+vt$$

(4) $$y=y_{0}\;$$

where *v* is the laser scanning speed. The default Heat Transfer in Solids module was used, with the initial temperature set to 293 K.

An Effective Powder Thermal Conductivity *k_powder_* Was Estimated Using a Porosity‐ and Composition‐based Mixing Approach According to Equations (5) and (6) [46],

(5) $$k_{powder}=\left(1-\varphi \right)k_{eff}$$

(6) $$k_{eff}=\psi k_{1}+\left(1-\psi \right)k_{2}$$

where *φ* is the powder bed porosity, *ψ* is the weight fraction of the constituent materials, and *k_1_* and *k_2_* are their respective thermal conductivities [46].

The effective heat capacity *C_p_* was calculated according to Equation (7),

(7) $$C_{p}=\frac{2}{\left(\rho_{1}+\rho_{2}\right)}\;\left[\psi \rho_{1}C_{1}+\left(1-\psi \right)\rho_{2}C_{2}\right]$$

where *ρ_1_* and *ρ_2_* are the densities, and *C_1_* and *C_2_* are the specific heat capacities of the two materials.

The simulated two‐material composite consisted of a carbonaceous matrix and a eutectic NaCl/KCl salt mixture. Carbonaceous materials were selected instead of the protein precursor because thermal pre‐treatment converts hemoglobin into a phenol‐rich partially aromatized carbon‐rich material [13]. For this preliminary model, material properties were assumed constant during irradiation. While dynamic evolutions in thermal and optical properties during pyrolysis would improve accuracy, such efforts were beyond the scope of this initial modeling effort. A summary of all parameters, variables, and material properties used in the simulations is provided in Table S2.

### 4.5 Supercapacitor Fabrication

For electrochemical testing, pairs of identical square‐shaped GAs were printed by raster scanning an 8 mm × 8 mm area with a hatch spacing of 2 mm. The GAs were placed in a glass container and electrically connected to an external circuit using rigid medical‐grade stainless steel metal leads. The leads were mounted on a 3‐axis *xyz* stage and brough into contact with the top (laser‐irradiated) surface of each electrode. The electrodes were aligned planar and parallel to one another with a controlled spacing of ∼1 mm. For the electrolyte, a 1 M H_2_SO_4_ solution was used. Drying was found to decrease electrolyte wettability; therefore, washed wet GAs were directly used as electrodes, and the electrolyte was gradually drop‐cast until both electrodes were fully submerged (Figure 4a). The active mass of each electrode was measured post‐electrochemical testing by rinsing in deionized water until neutral pH, followed by drying to remove residual moisture.

### 4.6 Supercapacitor Characterization

CV, CP, and EIS measurements were conducted using an Interface 1000 potentiostat (Gamry Instruments Inc., USA). CV curves were obtained at scan rates of 5, 10, 20, 50, and 100 mV s^−1^, while CP curves were obtained at current densities of 0.5, 1.0, 2.0, 3.0, 4.0, 5.0, and 7.5 A g^−1^. Absolute capacitance *C_A_* was calculated from the CV curves according to Equation (8),

(8) $$C_{A}=\frac{\int_{V_{i}}^{V_{f}}I\left(V\right)\;dV}{2\times v_{s}\times \left(V_{f}-V_{i}\right)}\;$$

where *v_s_* is the scan rate, *V_f_* and *V_i_* are the potential limits, and *I(V)* is the voltametric current. The specific gravimetric capacitance was obtained by normalizing *C_A_* by the active mass. CV and CP data were collected after three stabilization cycles, with the fourth cycle plotted for analyses. Cycle stability measurements were conducted after ten stabilization cycles.

EIS measurements were conducted over a frequency range of 0.1 Hz to 1 MHz, and Nyquist plots were generated from the acquired measurements. Ragone plots were prepared by calculating the gravimetric energy density and power density from CP curves measured at different current densities. The energy density *E_SC_* was determined according to Equation (9),

(9) $$E_{SC}=\frac{1}{3600*m_{A}}\;\int_{t_{i}}^{t_{f}}iV_{o}dt$$

where *i* is the discharge current density, *V_o_* is the discharge potential window after the voltage drop, and *t_i_* to *t_f_* are the start and end times of discharge. Moreover, the corresponding power density *P_SC_* was calculated according to Equation (10).

(10) $$P_{SC}=3600\times \frac{E_{SC}}{t_{f}-t_{i}}$$

## Author Contributions

S.H. and C.B.A. conceived and designed the experiments. S.H. conducted the laser processing experiments. E.S. conducted the furnace processing experiments. S.H., A.D., M.R., E.S., and J.M. carried out material characterizations. S.H. and A.D. conducted the simulations. The manuscript was written by S.H. and C.B.A. with contributions from all authors. M.L.S. and C.B.A. supervised the project. All authors contributed to discussions, reviewed and revised the manuscript, and approved the final version.

## Conflicts of Interest

The authors declare no conflicts of interest.

## Supporting information




**Supporting File**: adma73352‐sup‐0001‐SuppMat.docx.

## Data Availability

The data that support the findings of this study are available from the corresponding author upon reasonable request.
