# Supplementary material for: Reactive Laser Additive Manufacturing of Hierarchically Structured Aerogels
Source: Adv Mater. 2026 May 8;38(33):e73352. doi: 10.1002/adma.73352 (PMC13261388; doi:10.1002/adma.73352)
Supplement: Supplementary file 1 — Supporting File: adma73352‐sup‐0001‐SuppMat.docx. [file ADMA-38-e73352-s001.docx]

Reactive laser additive manufacturing of hierarchically structured aerogels

Shuichiro Hayashi^1,2^, Ankit Das^1,2^, Marco Rupp^1,2^, Elizabeth Stump^1,2^, Joshua Miller^3^,
Michele L. Sarazen^3^, and Craig B. Arnold^1,2,*^

^1^Princeton Materials Institute, Princeton University, Princeton, NJ 08540, USA

^2^Department of Mechanical and Aerospace Engineering, Princeton University, Princeton, NJ 08540, USA

^3^Department of Chemical and Biological Engineering, Princeton University, Princeton, NJ 08540, USA

^*^Corresponding author email: cbarnold@princeton.edu

# **ADDITIONAL RESULTS**


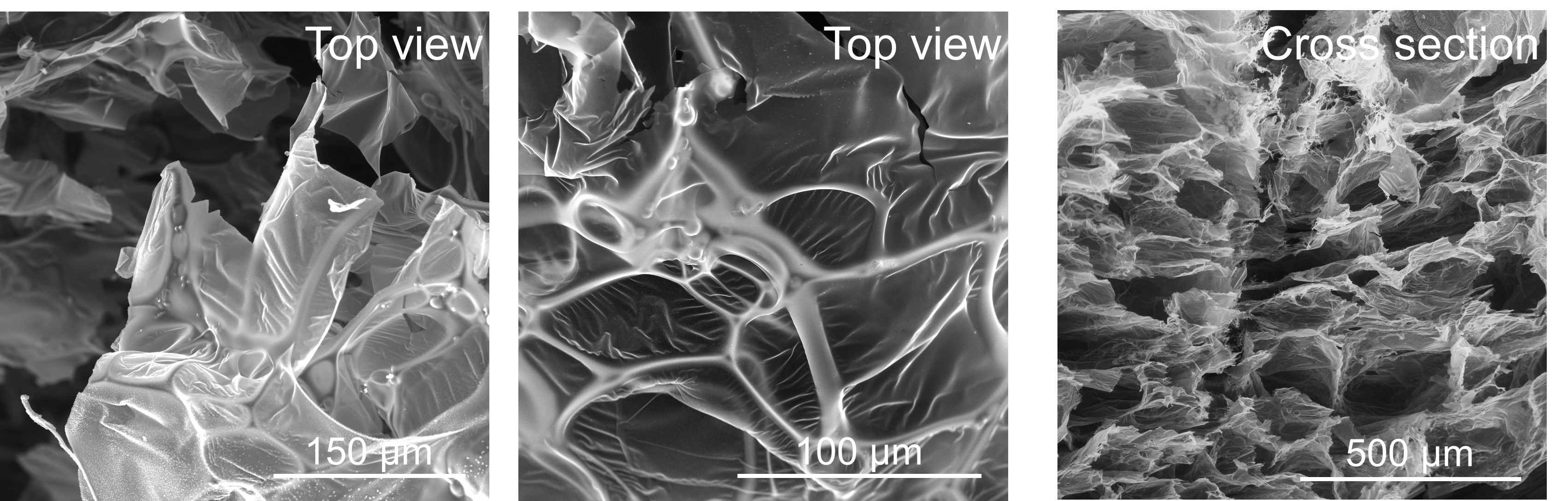


**Figure S1.** SEM images of the surface and cross-section of the laser printed GAs without salt additives (0 wt%) at different locations (N_2_). The salt-free GAs possesses a unique architecture consisting of interconnected ultrathin sheets and denser strut-like boundaries, comparable with those reported for furnace-fabricated protein-based aerogels.


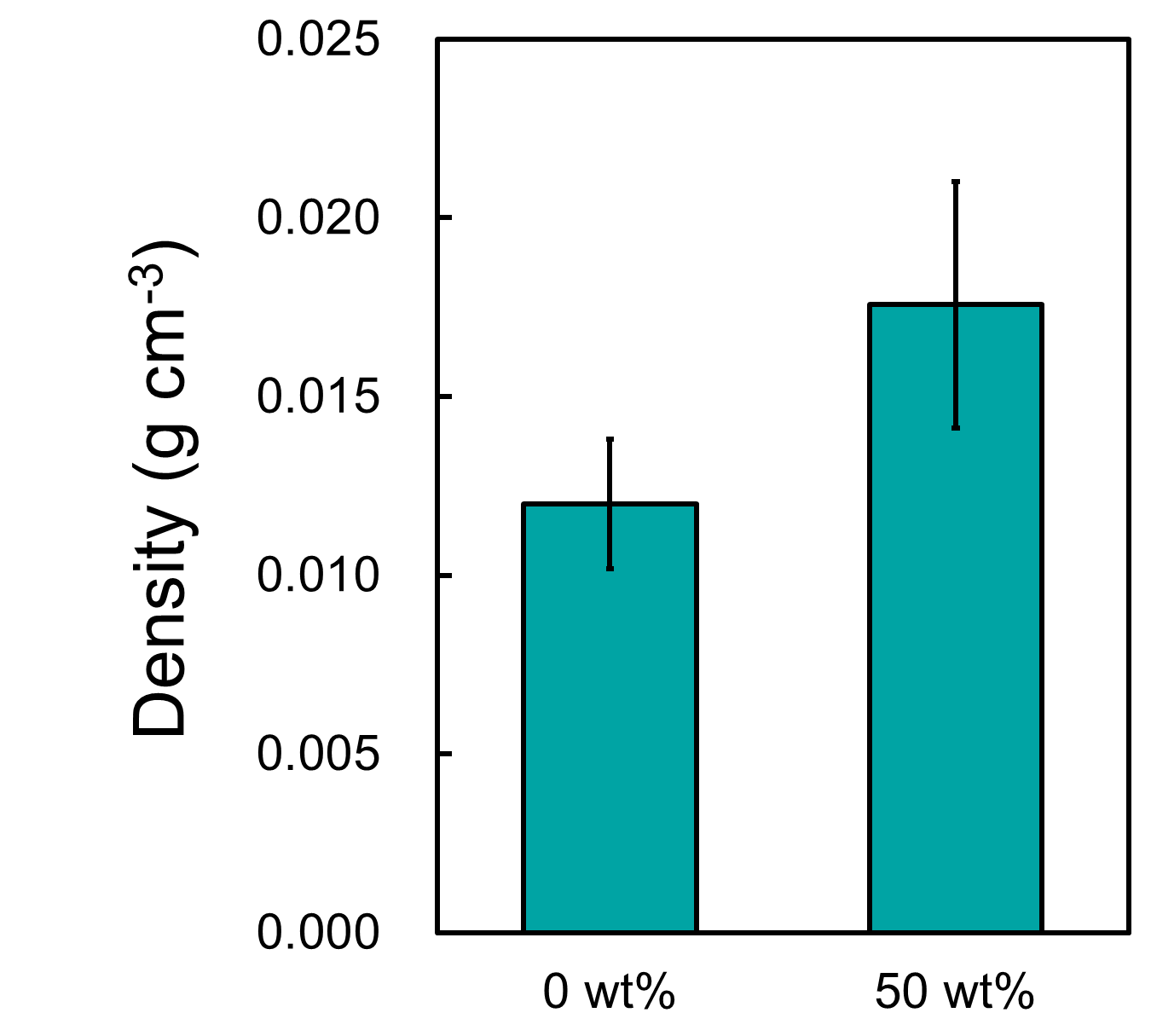


**Figure S2.** Measured volumetric densities of laser printed square GAs without (0 wt%) and with salt additives (50 wt%). The values and error bars indicate the mean and one standard deviation, respectively (n = 5).


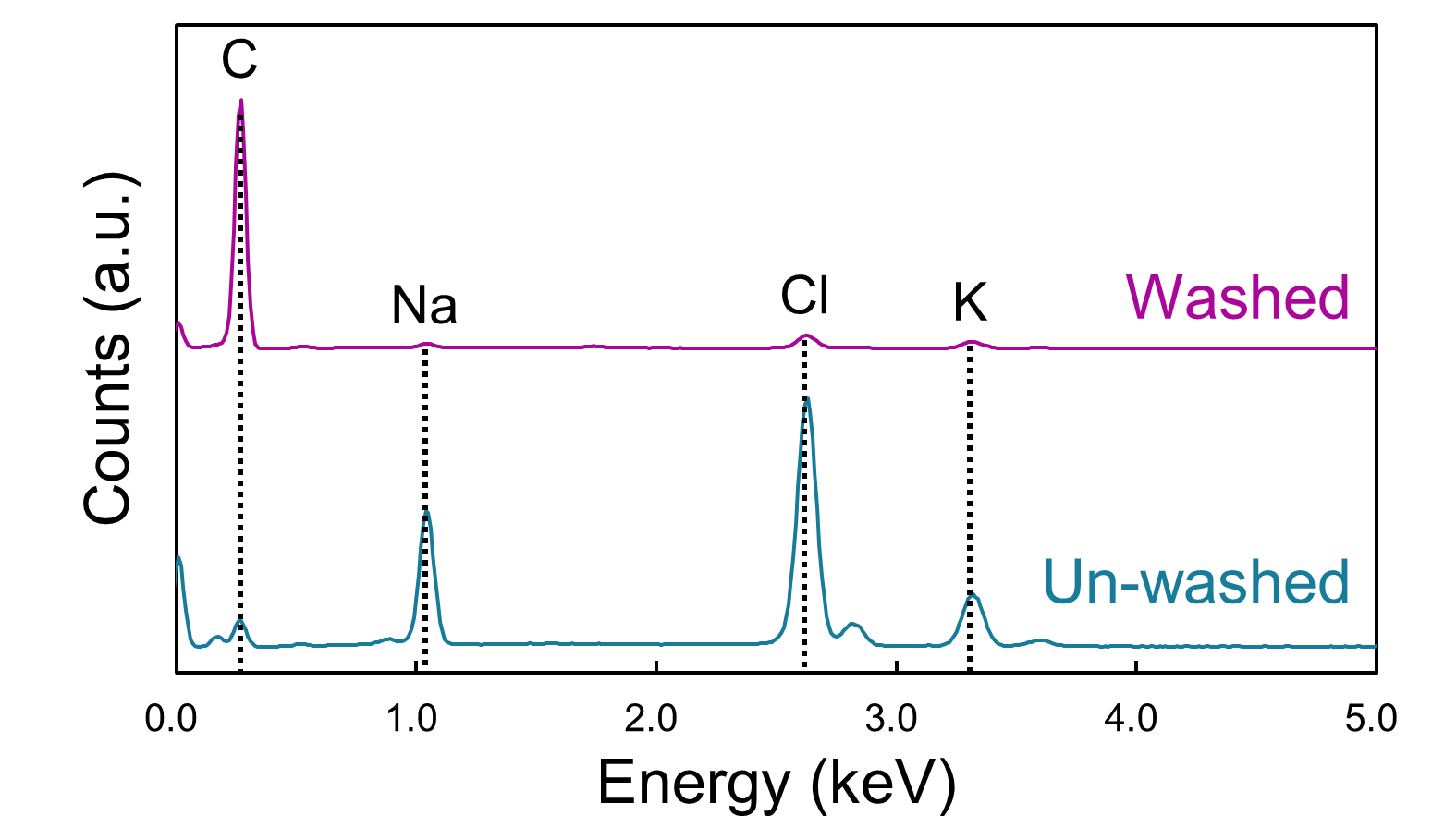


**Figure S3.** EDX spectra obtained from the surfaces of laser printed square GAs with salt additives (50 wt%) before and after water washing. EDX spectra for un-washed and washed were obtained from the field-of-views shown in **Figure 1e** and **g**, respectively. Significant decrease in intensity of the Na, Cl, K peaks indicate successful removal of salt crystallites from the surface through water washing. The notable increase in C peak intensity corresponds to the emergence of the underlying carbon-rich surface due to the removal of the top-layer of salt.


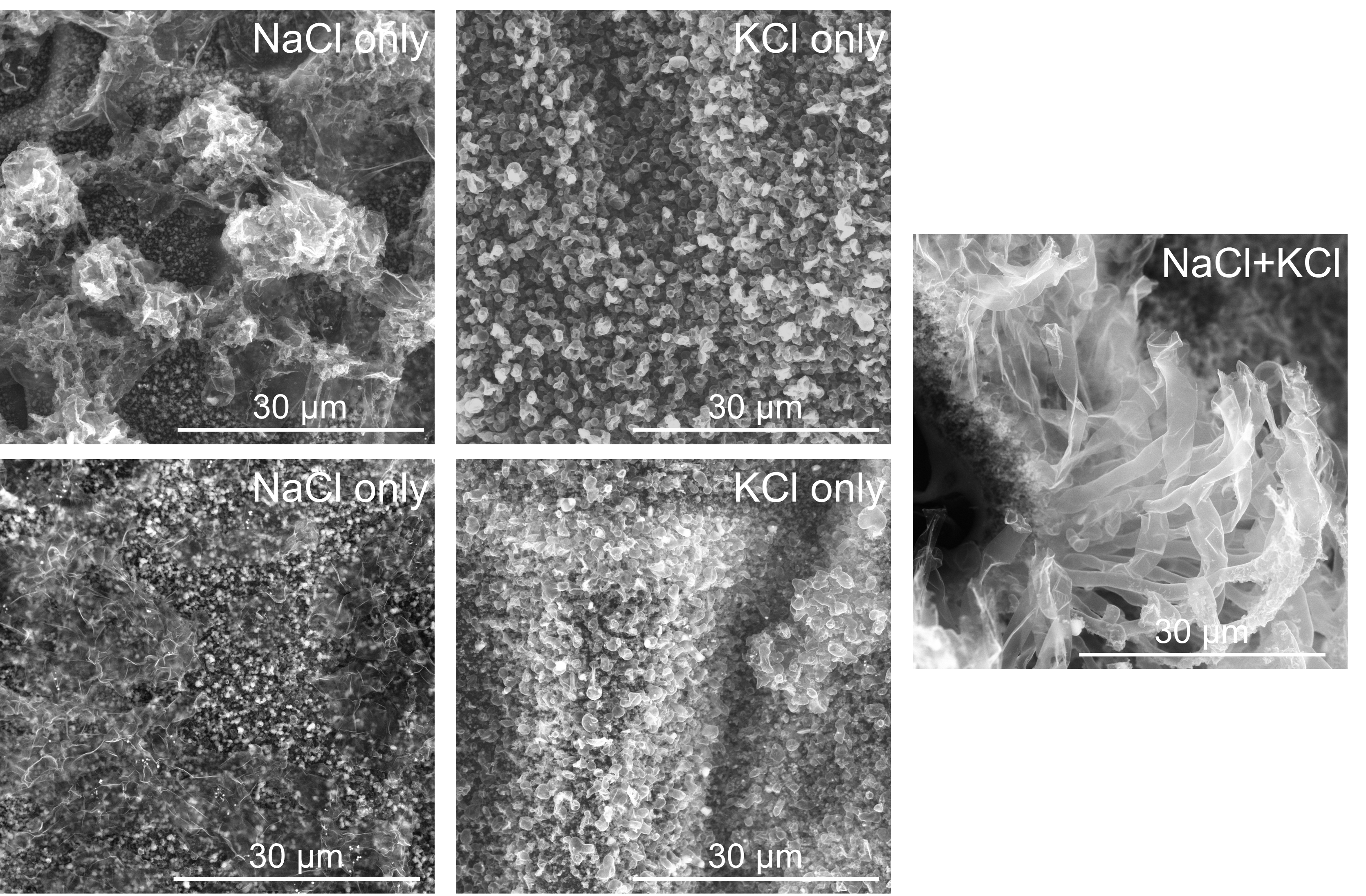


**Figure S4.** SEM images of the surface of the laser printed square GAs with salt additives (50 wt%) of different compositions: 50 wt% NaCl only, 50 wt%, KCl only, and 50 wt% eutectic mixture of NaCl and KCl. In the case of NaCl only, the formation of ultra-thin carbon films on the surface can be observed. On the contrary, in the case of KCl only, the formation of submicron or micron blisters across the surface can be observed. In the case of the eutectic salt mixture, evident formation of hollow tubular structures can be confirmed.


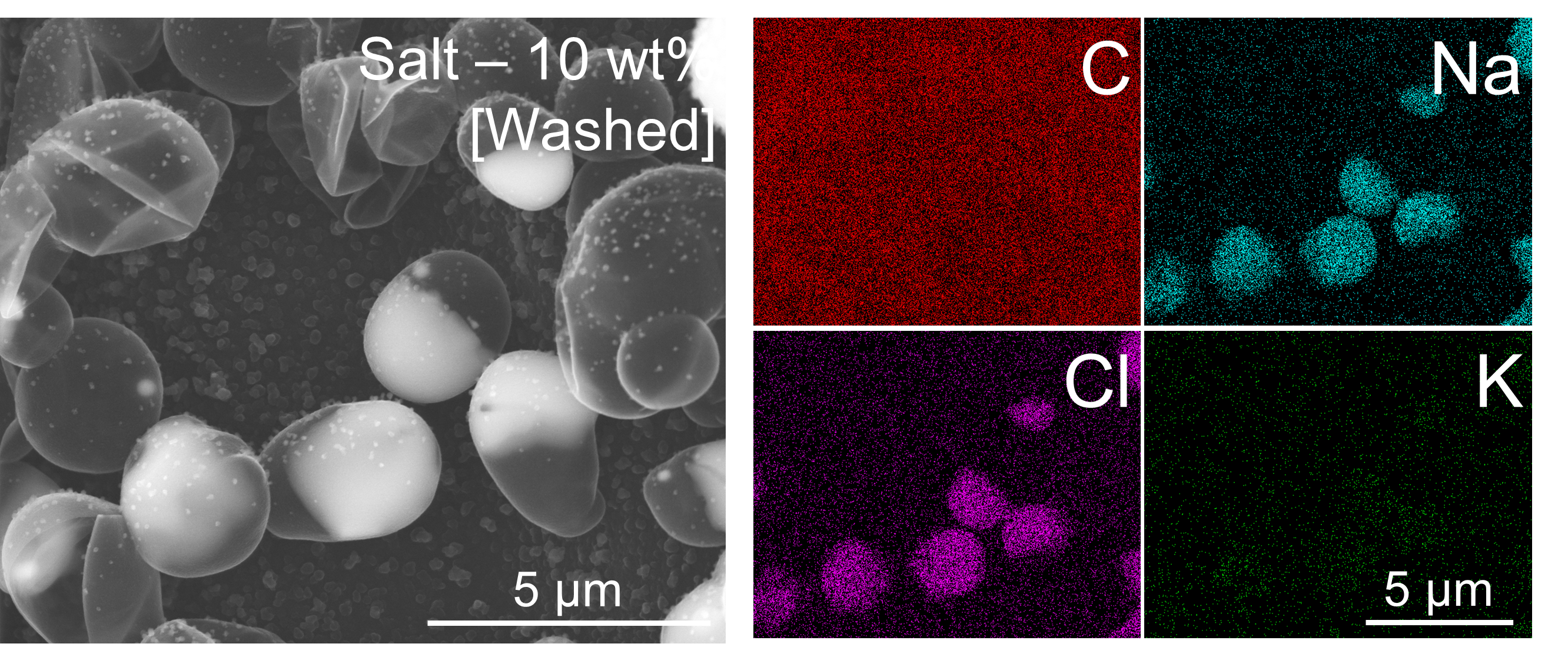


**Figure S5.** SEM image and corresponding elemental mapping images of the washed surfaces of GAs printed with 10 wt% salt concentration. Material with significantly brighter contrast is observed embedded within the microstructures indicating compositional differences (left SEM). EDX analysis confirms that these bright regions correspond to residual salt encapsulated within the structures, even after the current washing process.


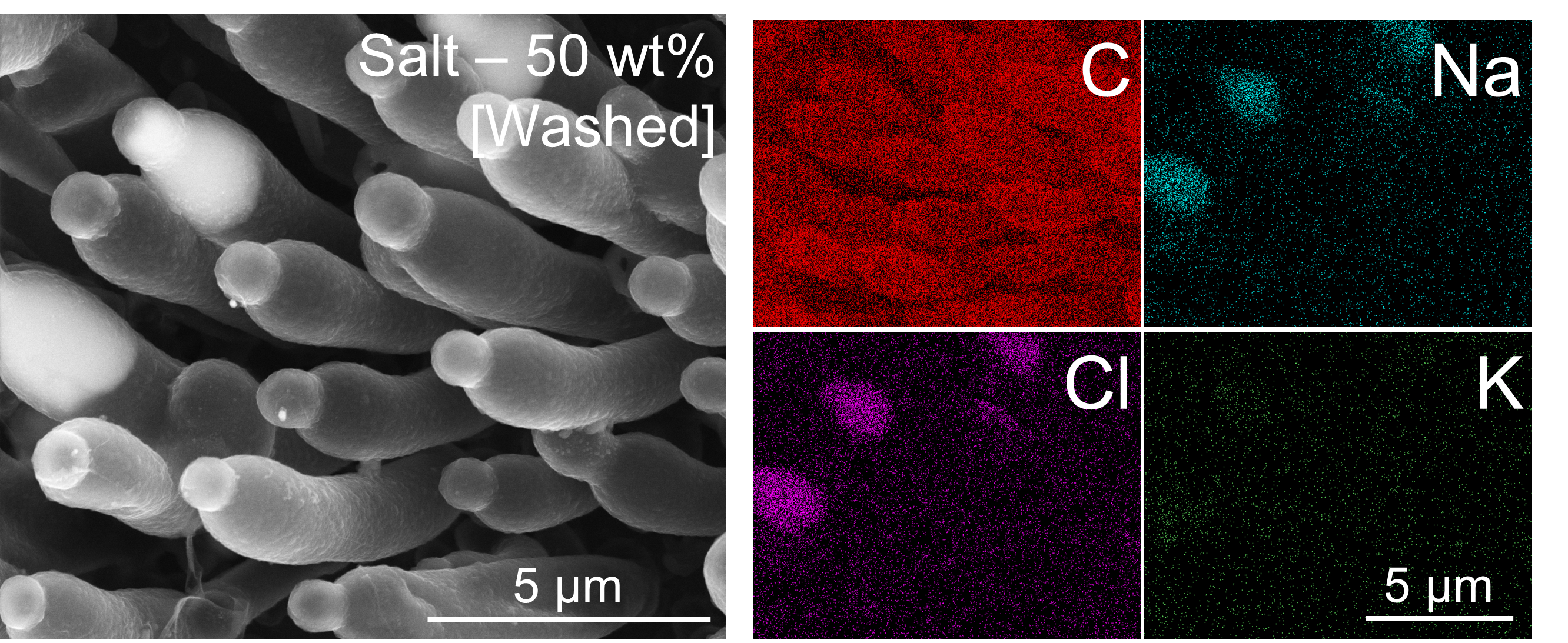


**Figure S6.** SEM image and corresponding elemental mapping images of the washed surfaces of GAs printed with 50 wt% salt concentration. Bright-contrast regions similar to those observed in **Figure S5** are observed. The slightly elevated volumetric density observed (**Figure S2**) may be attributed to the presence of these residual salts. This suggests that more prolonged or chemically intensive washing protocols may be necessary for complete removal, especially for tubular features with closed tips.


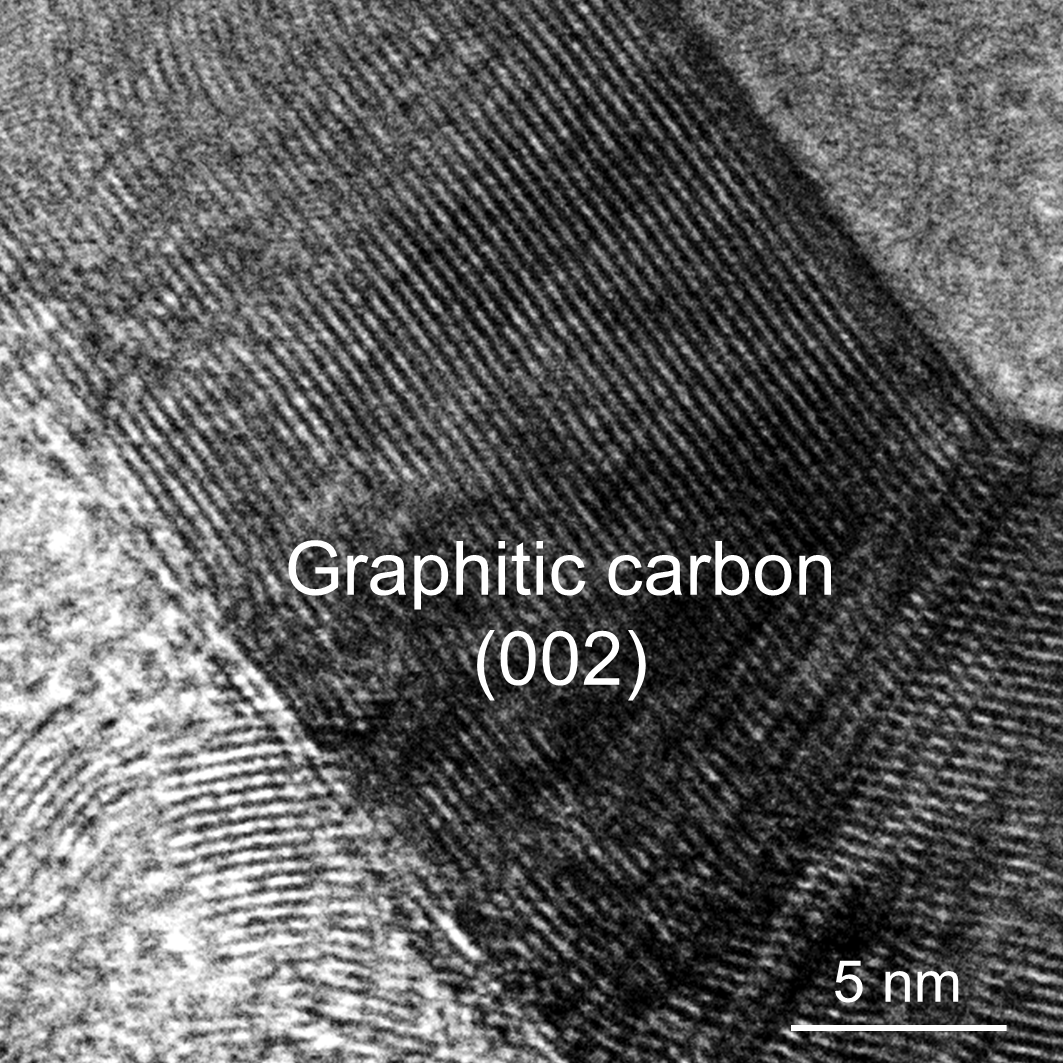


**Figure S7.** TEM image of the GA printed without salt additives (0 wt%). The 0 wt% structure exhibits linear multi-stacked lattice fringes, which corresponds to the (002) plane of graphitic carbon, with minimal in-plane defects and high out-of-plane crystallinity.


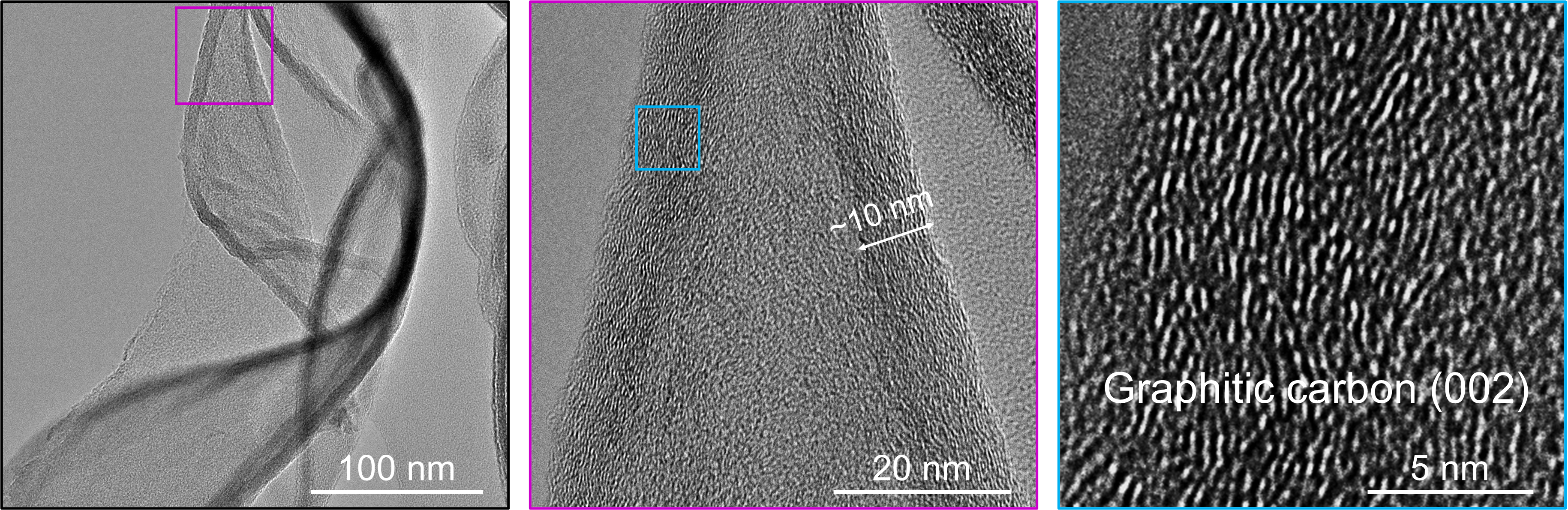


**Figure S8.** Progressively higher magnification TEM images of the edge of a microsphere formed with salt additives (10 wt%). The darker contrast along the edges suggests these features are three-dimensional spheres (left). Higher-magnification images of the microsphere edges shows that the walls are ~10 nm thick (middle) and composed of multilayered graphitic planes (right), revealing that the observed surface features are graphitic microspheres.


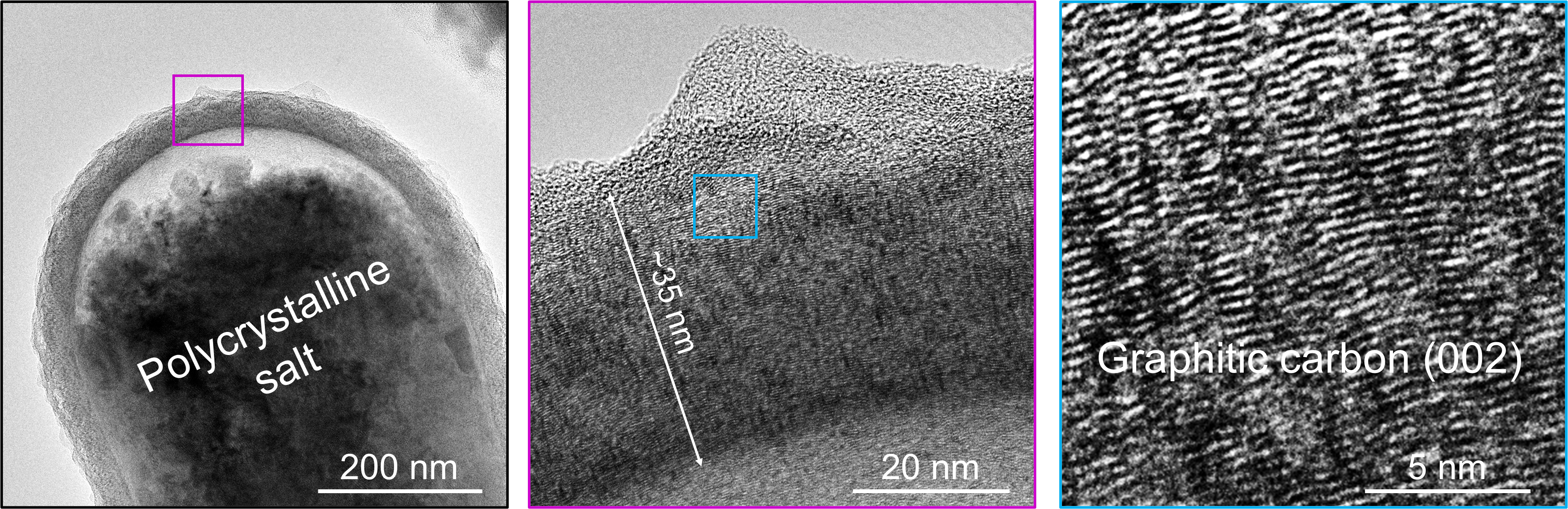


**Figure S9.** Progressively higher magnification TEM images of the tip of a closed-tipped microtube formed with salt additives (50 wt%). As in the case of the microspheres, the darker edge contrast suggests that these features are three-dimensional tubular structures. Similar to the graphitic microspheres observed at 10 wt% salt concentration, residual salt crystals are found entrapped within the microstructure (left). Consistent with SEM observations in **Figure S6**, the salt is primarily localized near the tip region. Moreover, the residual salt appears to exist as an aggregation of smaller crystallites (left). Higher-magnification TEM images of the tip (middle) reveal that the tubular walls are ~35 nm thick and consist of multilayered graphitic planes (right).


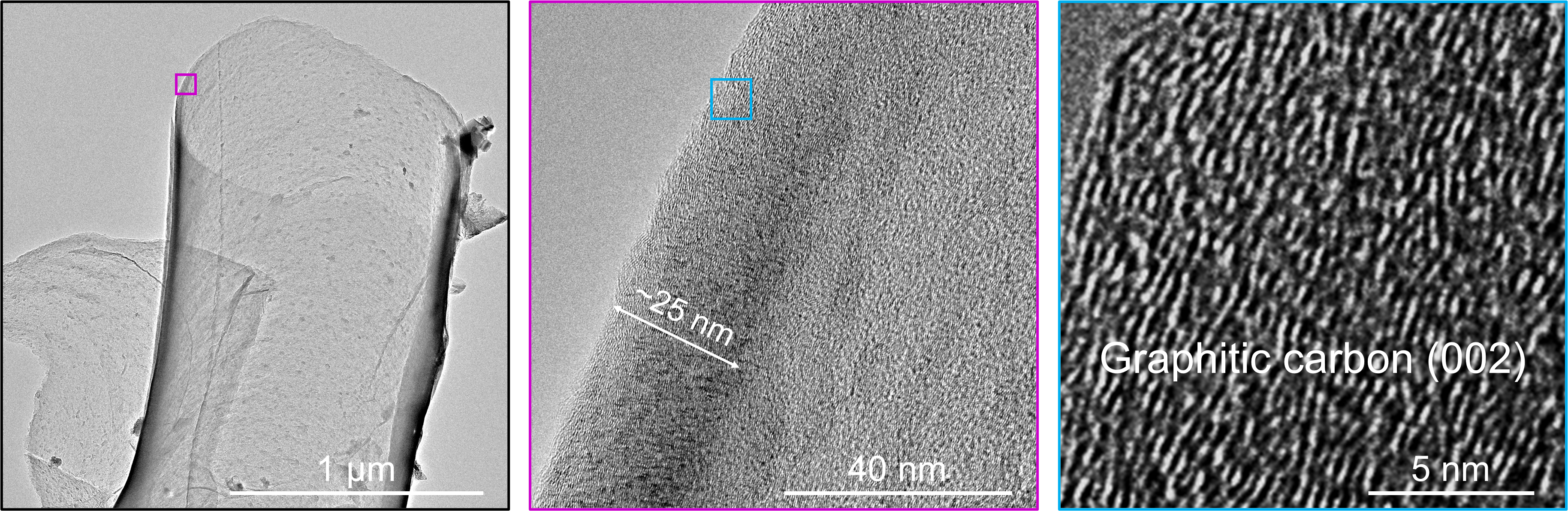


**Figure S10.** Progressively higher magnification TEM images of the tip of an open-tipped microtube formed with salt additives (50 wt%). These open-tipped microtubes exhibit comparable crystallinity but a slightly thinner wall thickness of ~20 nm (middle, right). The tip opening may be attributable to volatile salt extrusion facilitated by the thinner walls during rapid laser-induced heating.


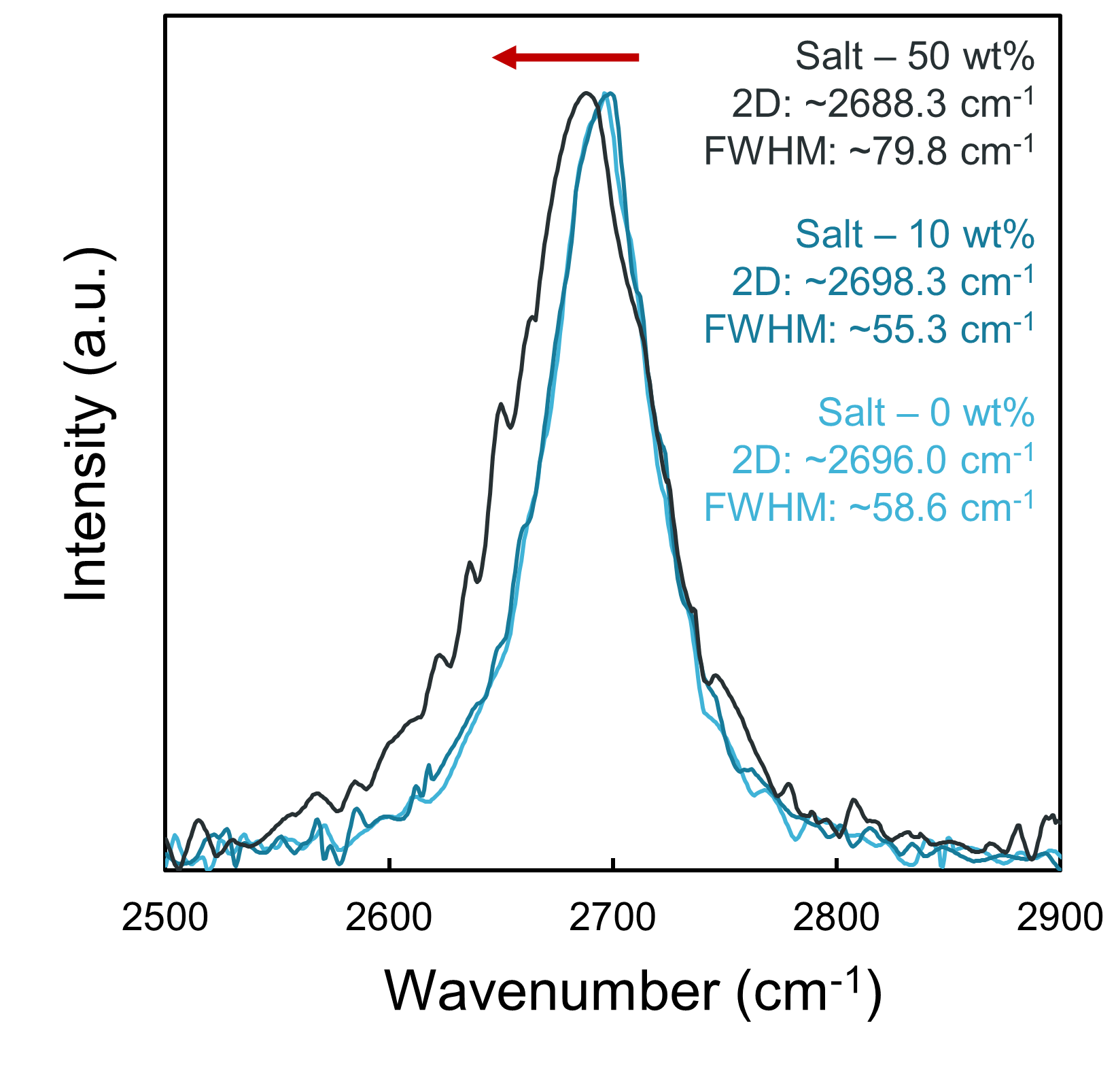


**Figure S11.** Normalized 2D band of the Raman spectra shown in **Figure 2h**.


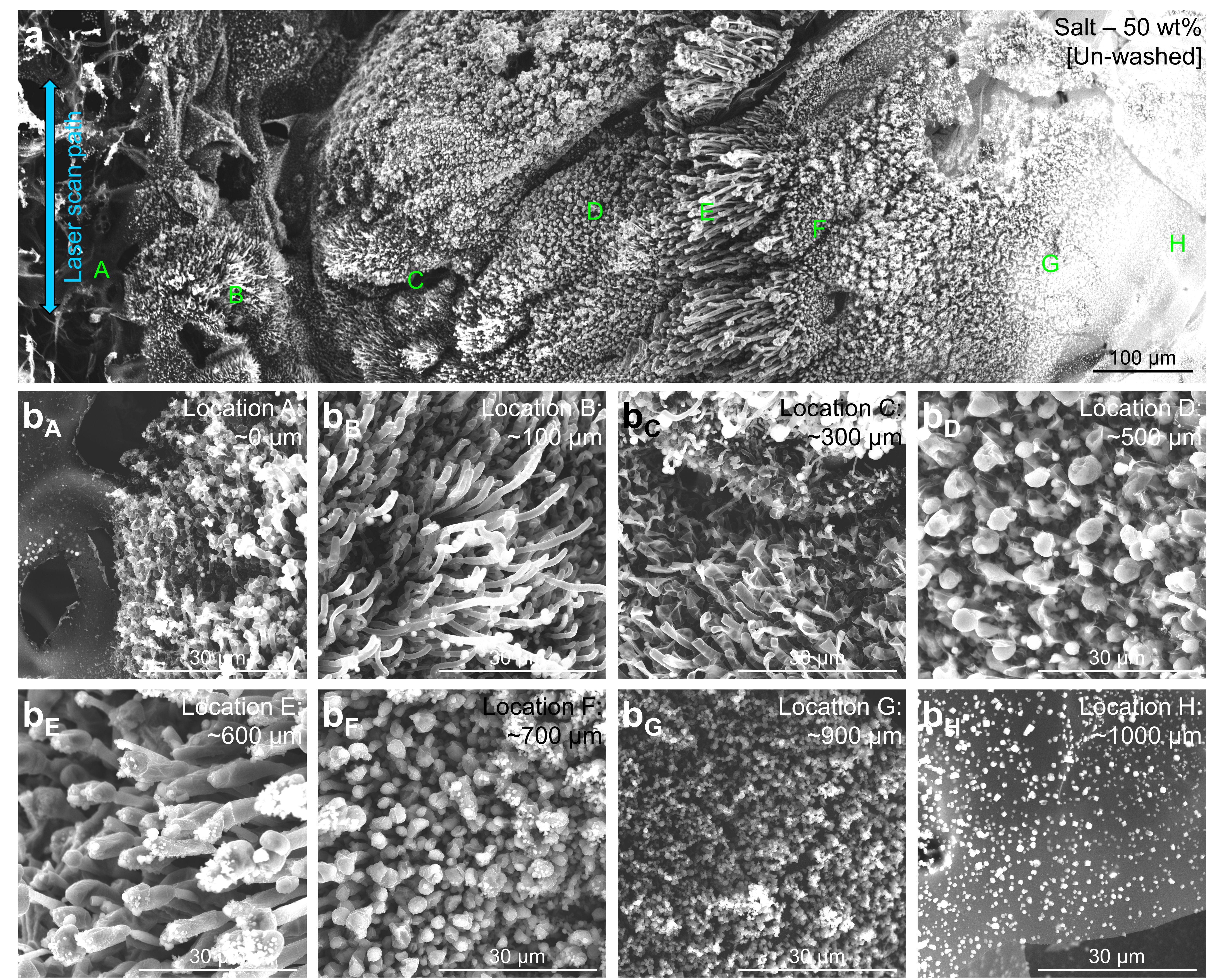


**Figure S12.** **a** SEM image of the edge of an aerogel printed with 50 wt% salt before water washing. **b** Higher-magnification SEM images for different distances from the laser scan path indicated in **a**. Additional discussions below.


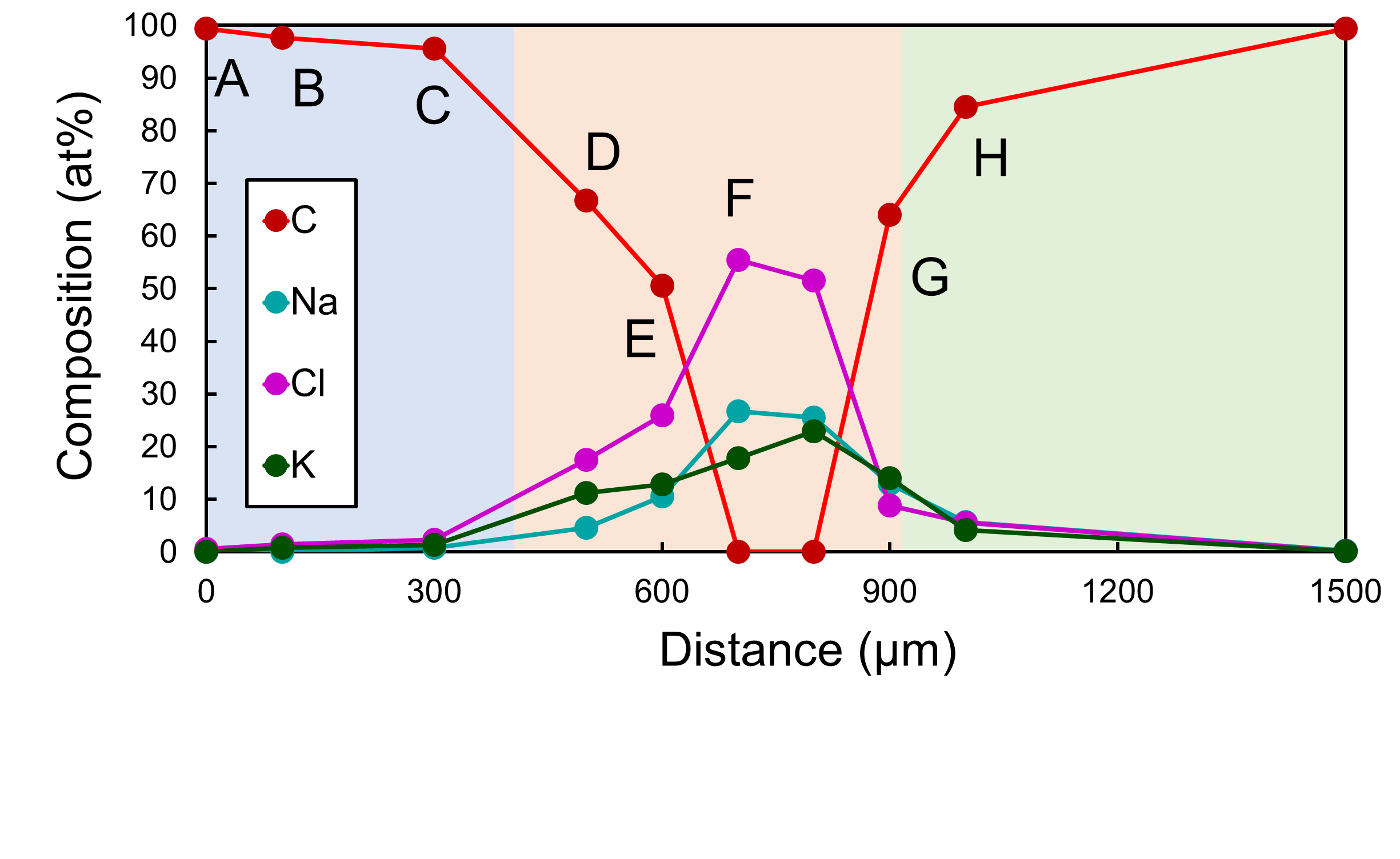


**Figure S13.** Elemental composition of the surface at different distances from the laser scan path. Additional discussions below.


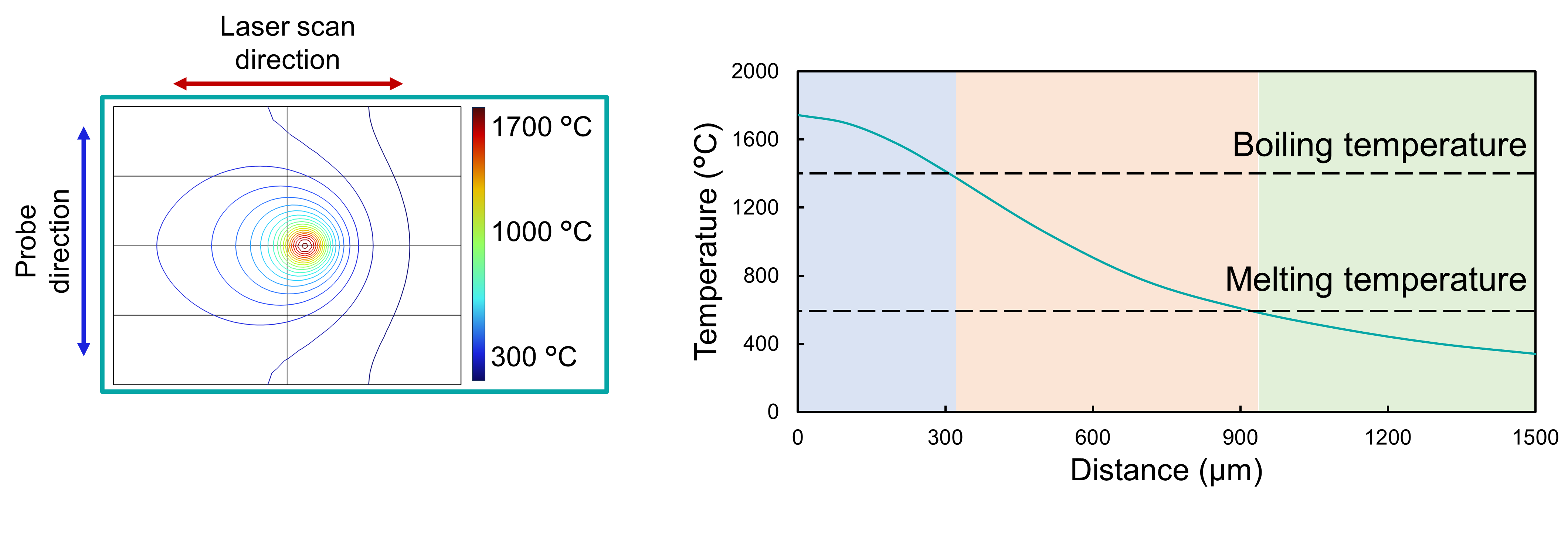


**Figure S14.** Simulated temperature gradient induced at the surface by laser processing. Additional discussions below.


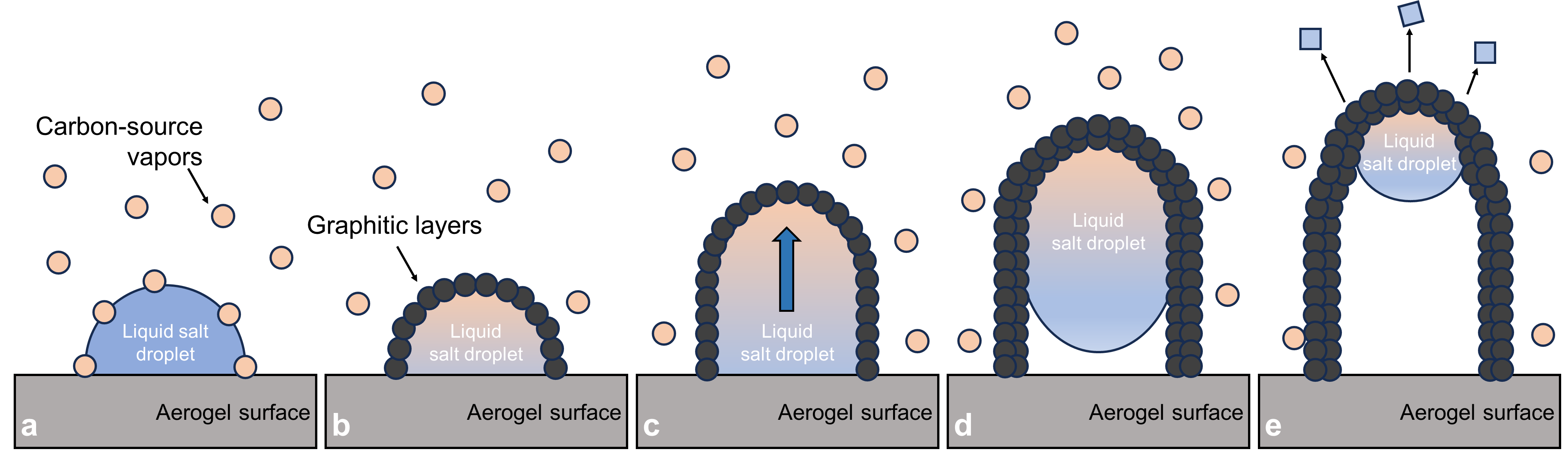


**Figure S15.** Schematic illustrating the transient multiphase environment during laser printing of GAs. **a** Upon laser irradiation salt crystallites melt into alkali halide droplets, serving as nucleation sites. **b** Carbon-rich vapors generated during pyrolysis precipitate onto the droplet surface, initiating graphitic layer growth. **c** Continued anisotropic deposition leads to formation of tubular structures, and **d** multilayered walls develop under sustained carbon flux and thermal gradients. **e** As the salt gradually vaporizes, droplet volume decreases, resulting in tip tapering and salt entrapment. Additional discussions below.


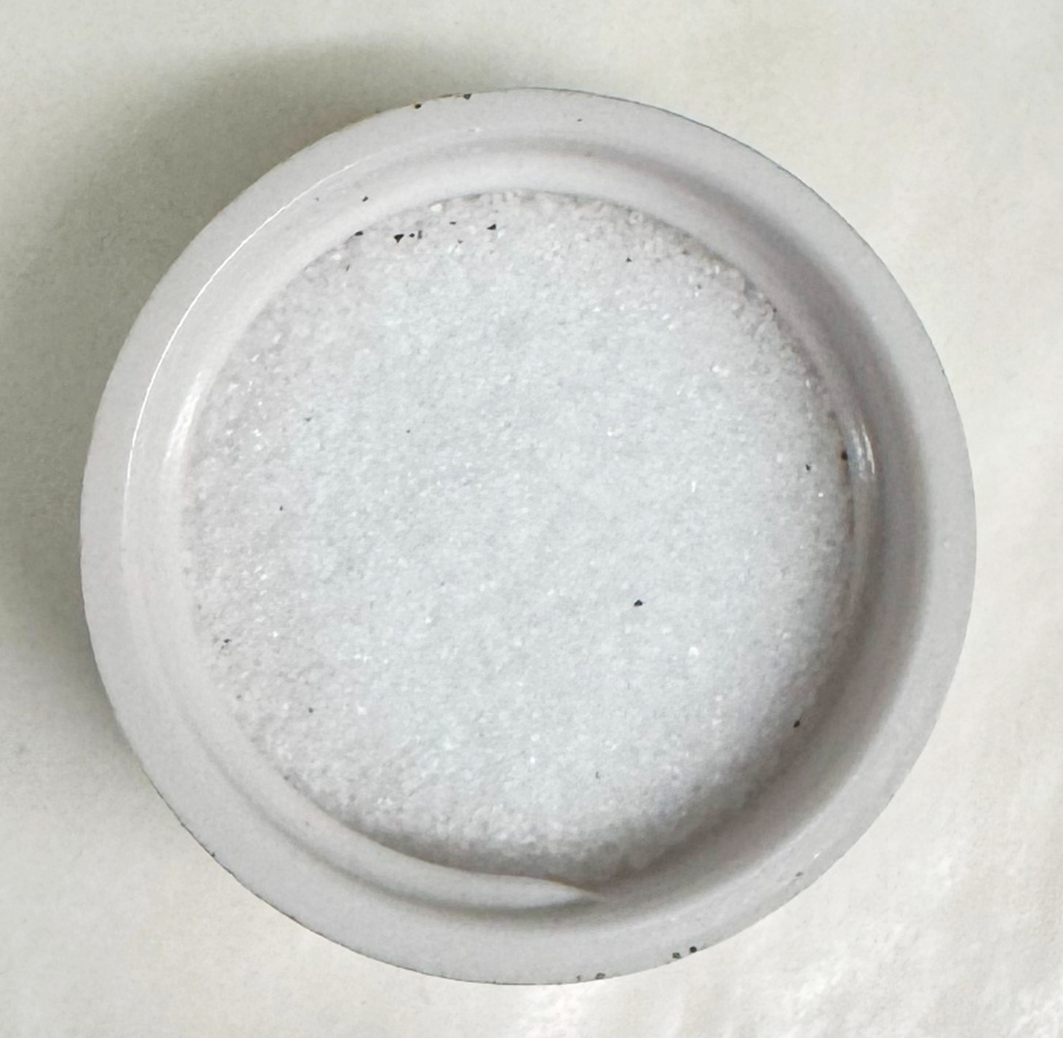


**Figure S16.** Optical image of a powder bed of NaCl/KCl salt mixture laser processed at a laser power of 10.0 W and a scanning speed of 2.00 mm s^-1^. After laser processing, no visible changes (e.g., melting) of the salt mixture were observed, indicating low optical absorbance.


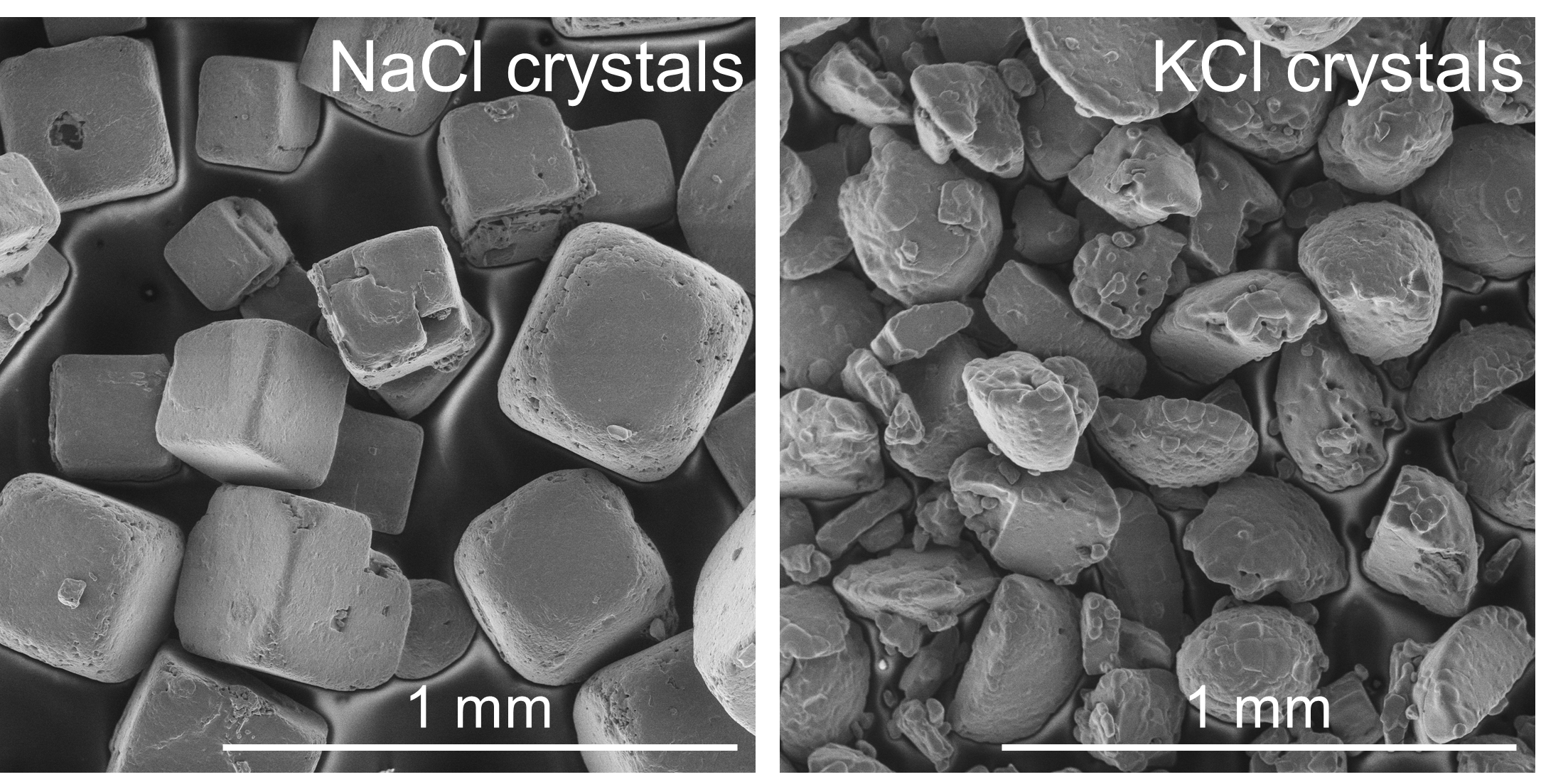


**Figure S17.** SEM images of the salt crystals before laser irradiation. The salts used in this study are couple hundred microns large in size prior to laser irradiation. As the salt crystallites observed via TEMs and SEMs indicate a smaller size, it is suggested that laser irradiation leads to their instantaneous vaporization due to the high peak temperatures induced at the laser focus, which will then precipitate as smaller crystallites on the surface of the aerogel.


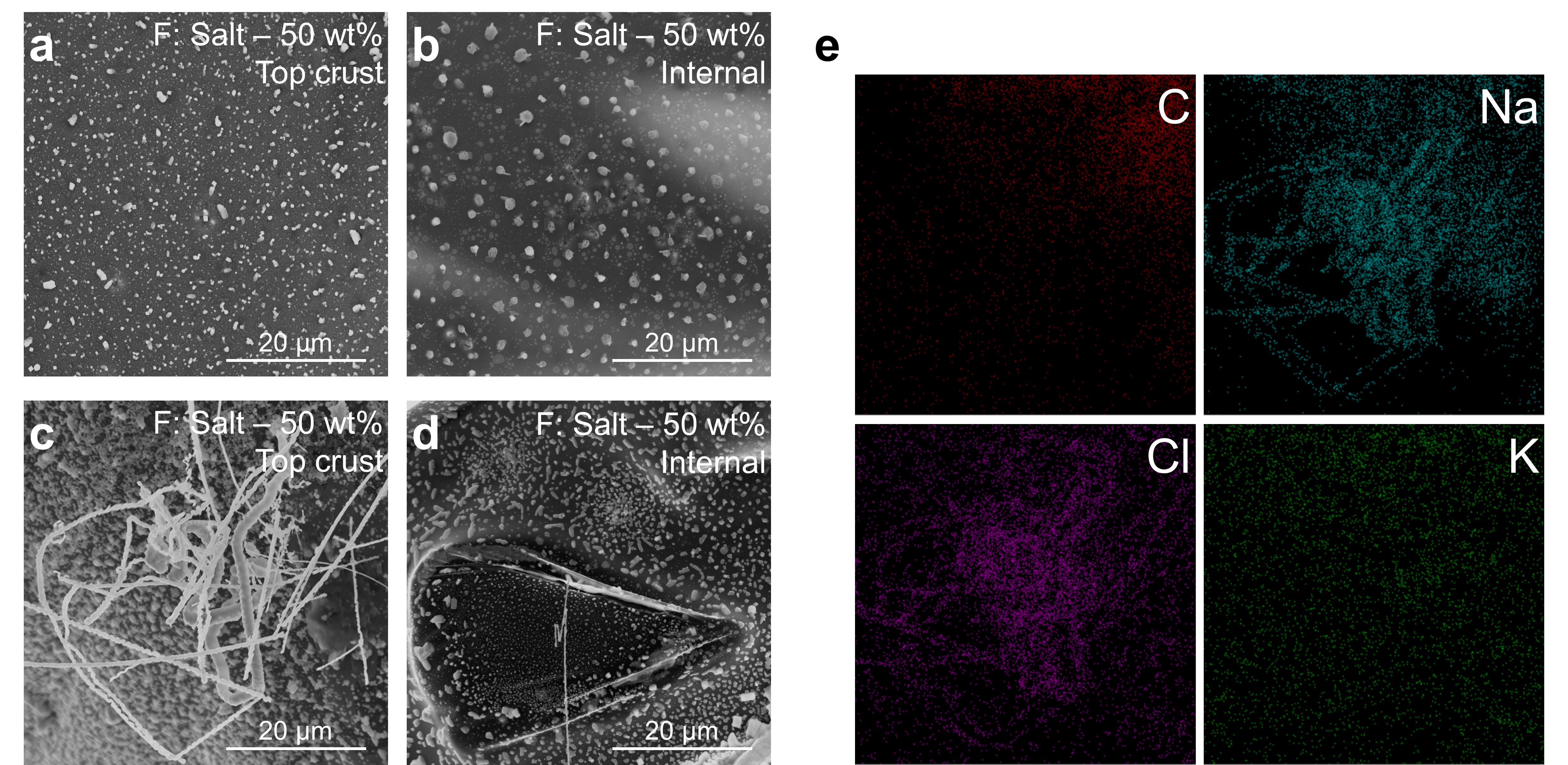


**Figure S18.** **a**–**d** SEM images of the top-surfaces and cross-sections of GAs prepared using a furnace via the methodology presented previously.^1^ **e** Elemental mapping images of the field-of-view shown in **c**.


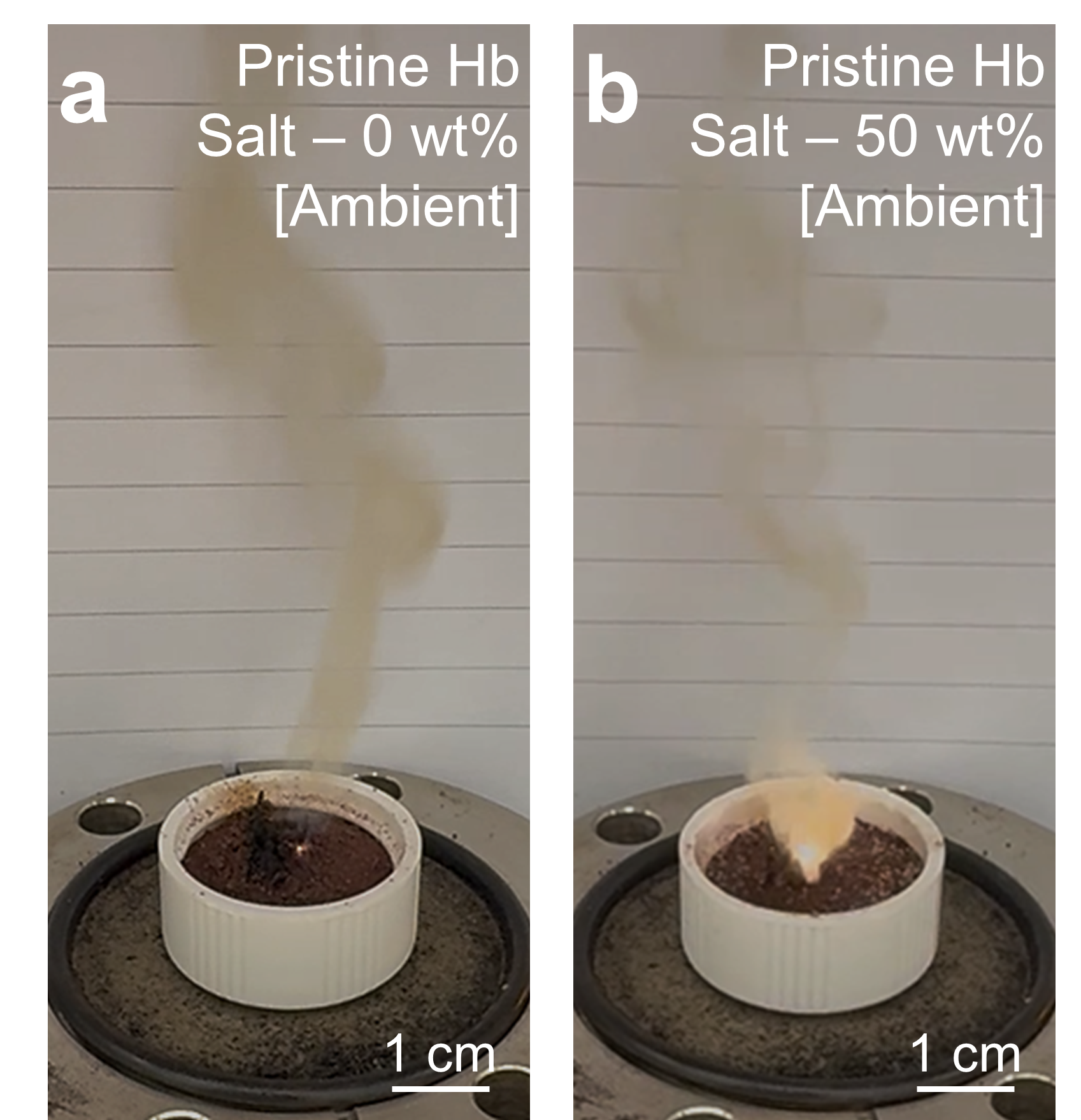


**Figure S19.** Photographs of the laser-printing process with pristine protein-based powder mixed with **a** 0 wt% and **b** 50 wt% salt under ambient conditions. Contrary to the case of pre-treated hemoglobin powder, the generation of dense dark vapors from the laser focal volume can be observed. Moreover, the light emission from laser processing was significantly dimmer.


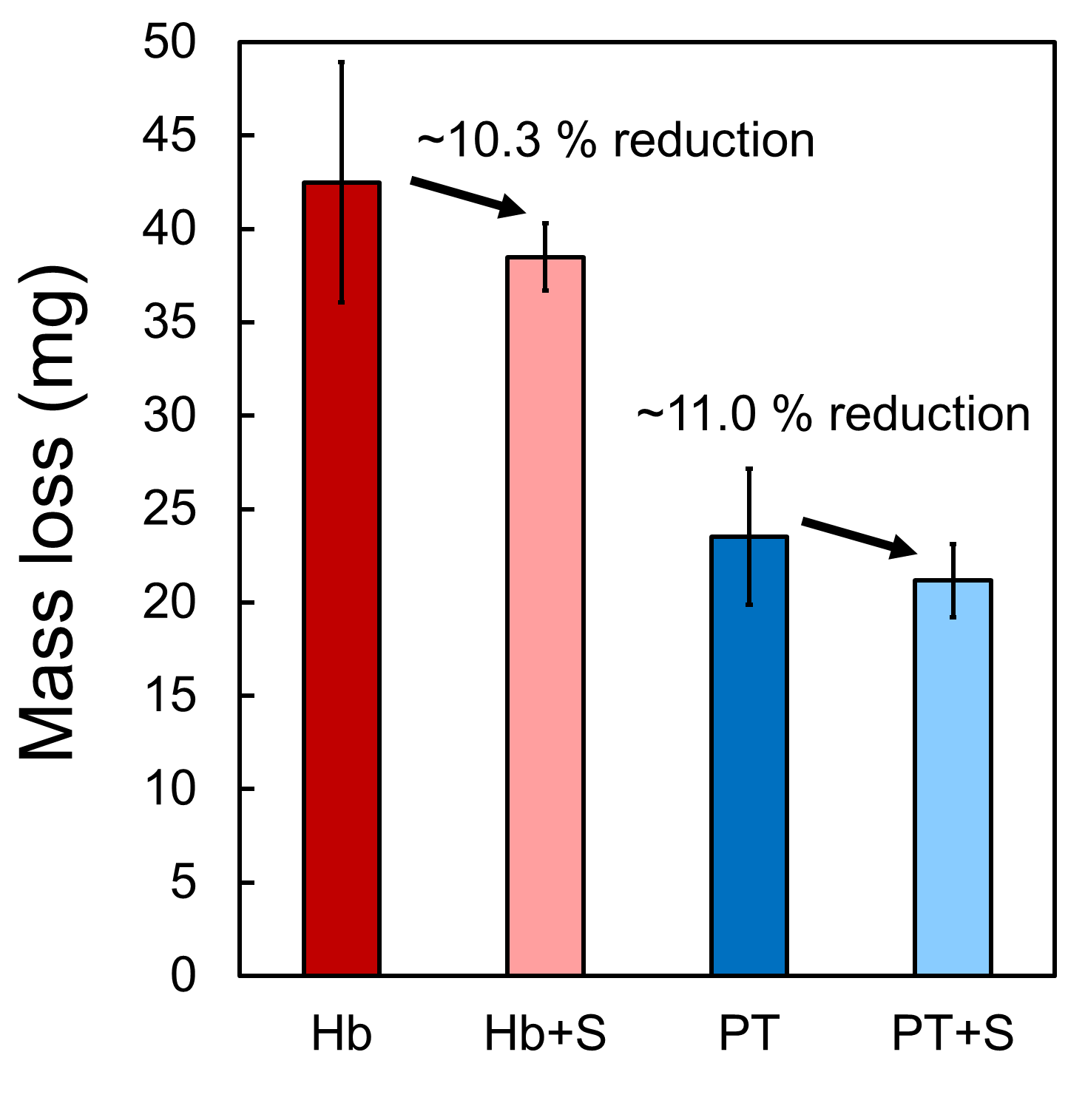


**Figure S20.** Mass loss of the powder bed following laser processing. Mass loss was calculated by comparing the total mass of the powder holder and powder bed before irradiation with the total mass of the powder holder, residual bed, and the printed structure (n = 5). Hb: pristine hemoglobin protein-based power without salt, Hb+S: pristine hemoglobin protein-based power mixed with 50 wt% salt, PT: pre-treated hemoglobin protein-based power, PT+S: pre-treated hemoglobin protein-based power mixed with 50 wt% salt.


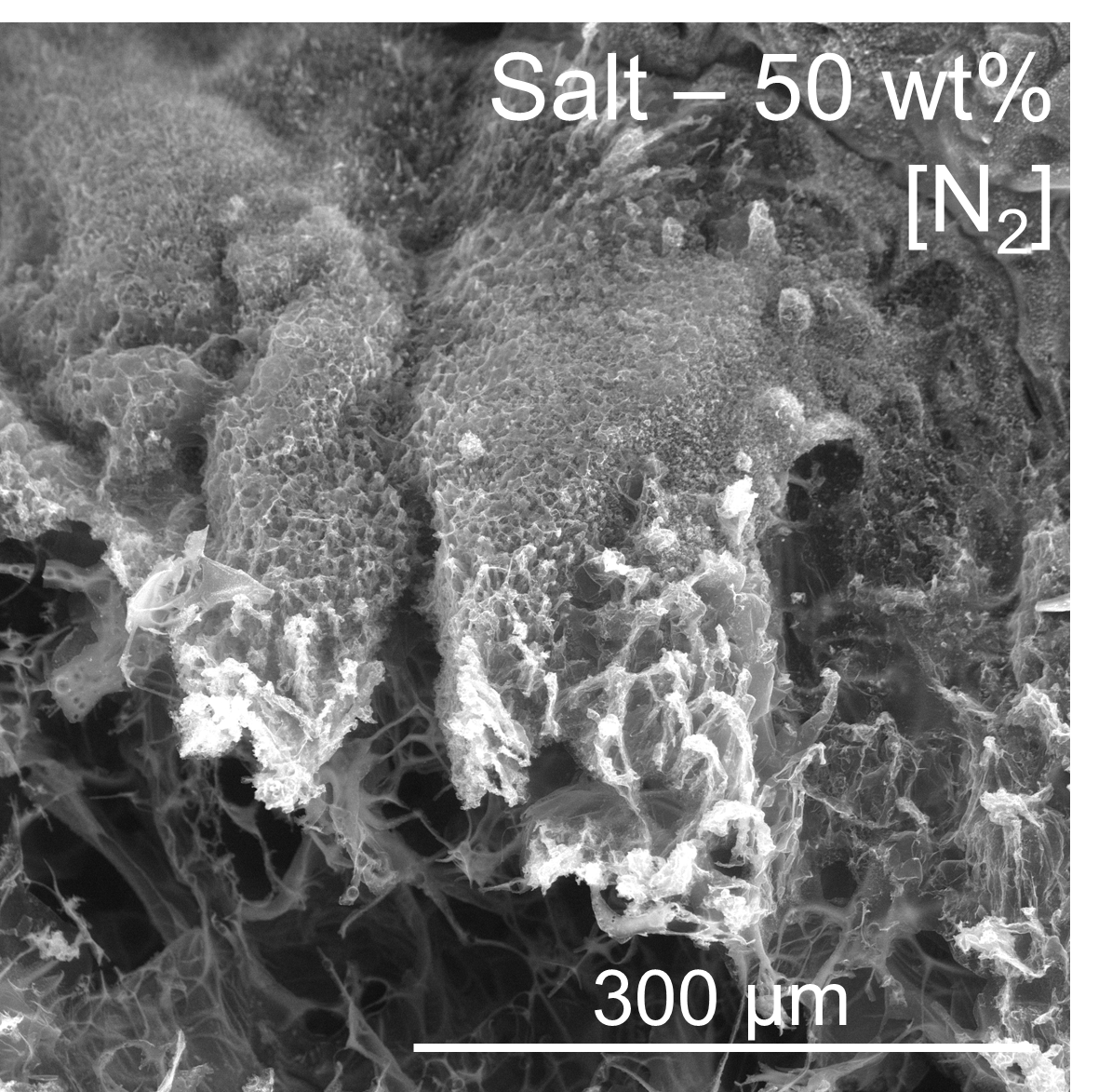


**Figure S21.** SEM image of the surface of the laser printed GA with salt additives (50 wt%) under inert N_2_ conditions. While the surface is covered in microscale tubules, the underlying aerogel framework is generally sheet-like.


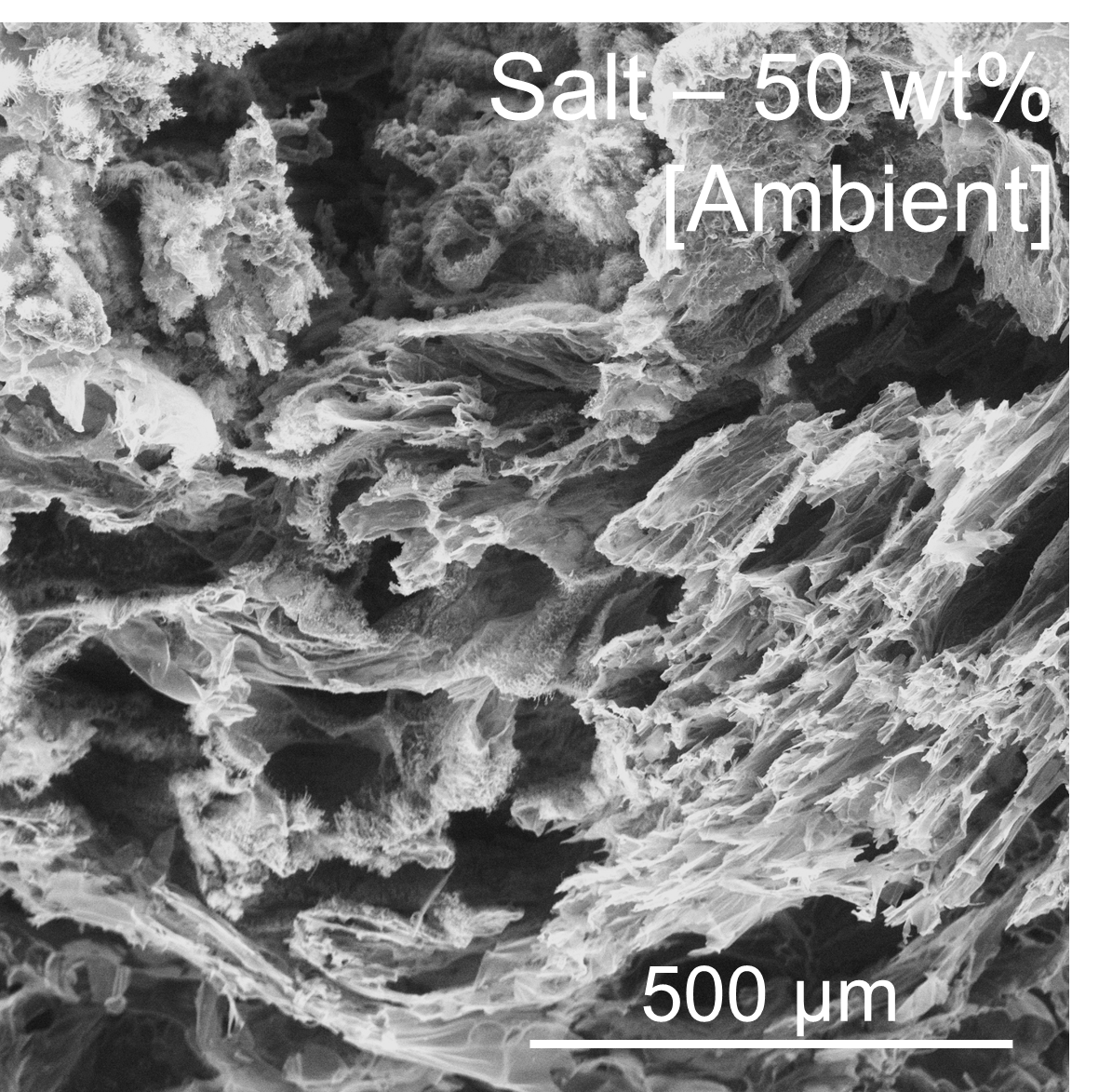


**Figure S22.** SEM image of the cross-section of the laser printed GA with salt additives (50 wt%) under ambient conditions. The underlying aerogel framework is macroporous, with pore diameters in a similar range as those for salt-free GAs printed under N_2_ conditions.


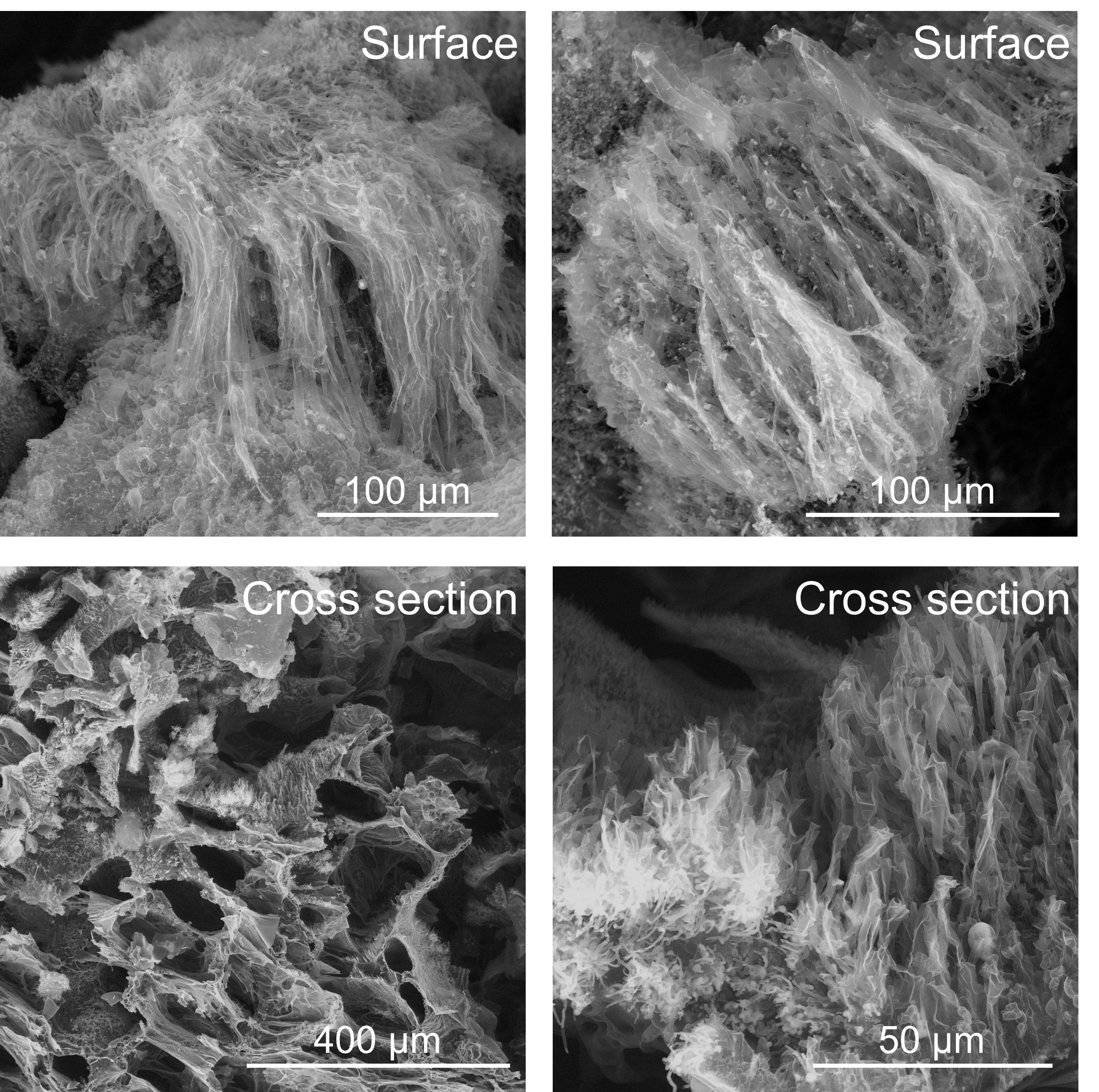


**Figure S23.** SEM images of the surface and cross-section of the laser printed GAs with salt additives (50 wt%) under ambient conditions. The salt-assisted GAs printed in ambient also possessed microscale tubular surface features externally (surface) and internally (cross-section).


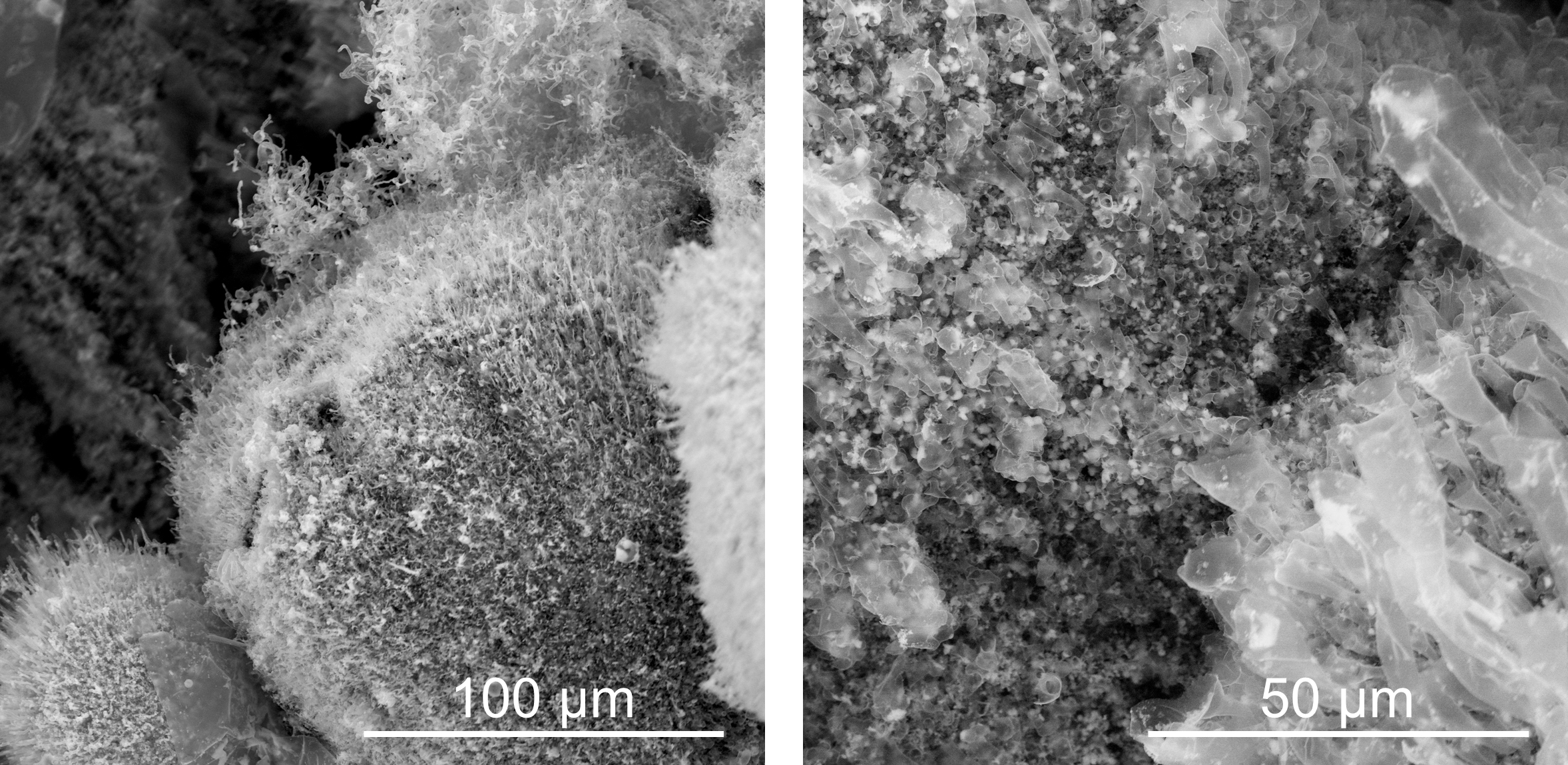


**Figure S24.** SEM images of the surface of the laser printed GA with salt additives (50 wt%) under ambient conditions. In addition to the microscale tubular surface features, the existence of smaller nano-scaled features and particle aggregates can be observed throughout the surface.


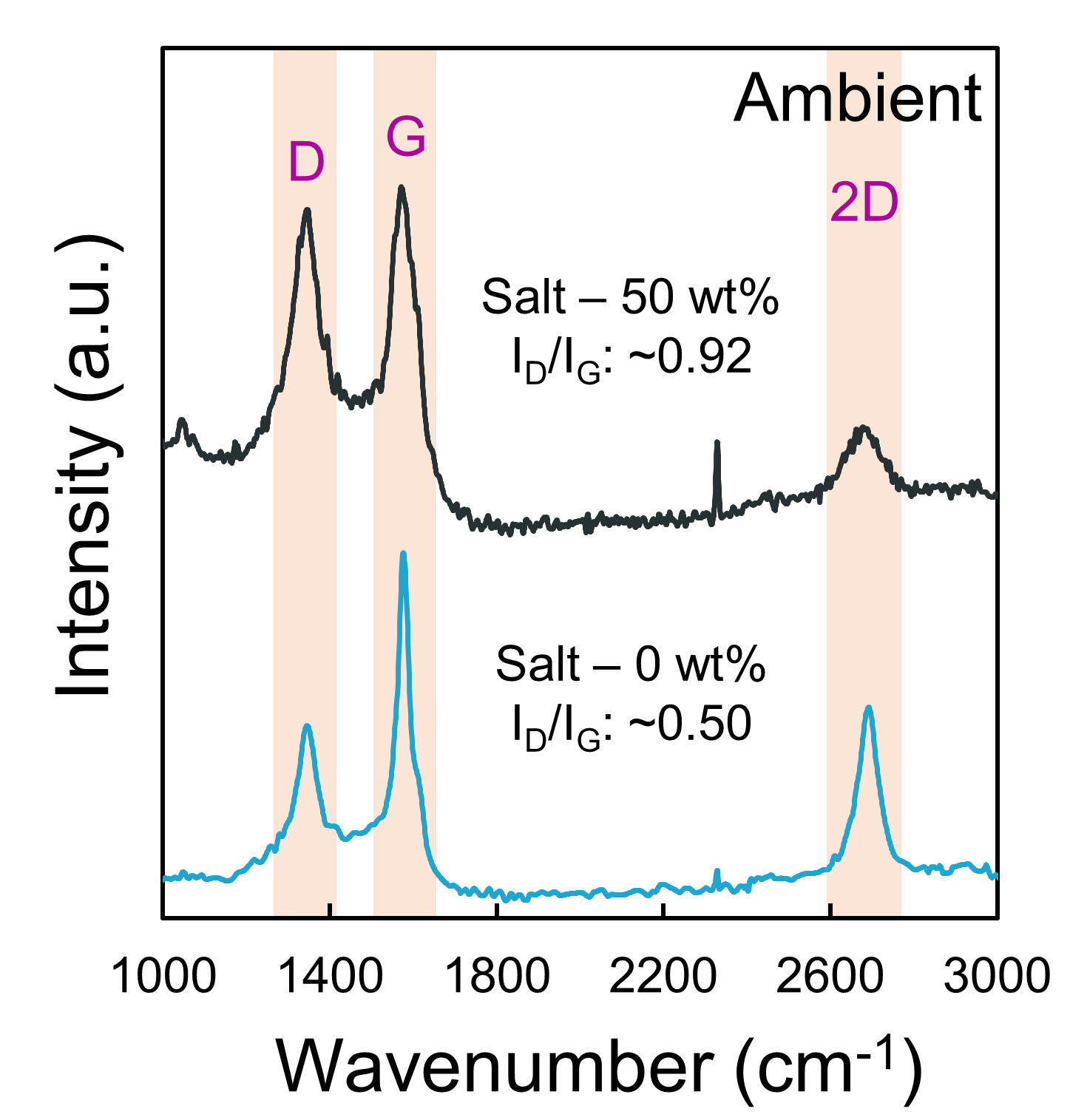


**Figure S25.** Raman spectra of aerogels printed with and without salt under ambient conditions. The significantly higher baseline peak of atmospheric CO_2_ (~2330 cm^-1^), may be a result of reduced overall Raman signal due to increased surface roughness for the salt-assisted GA.


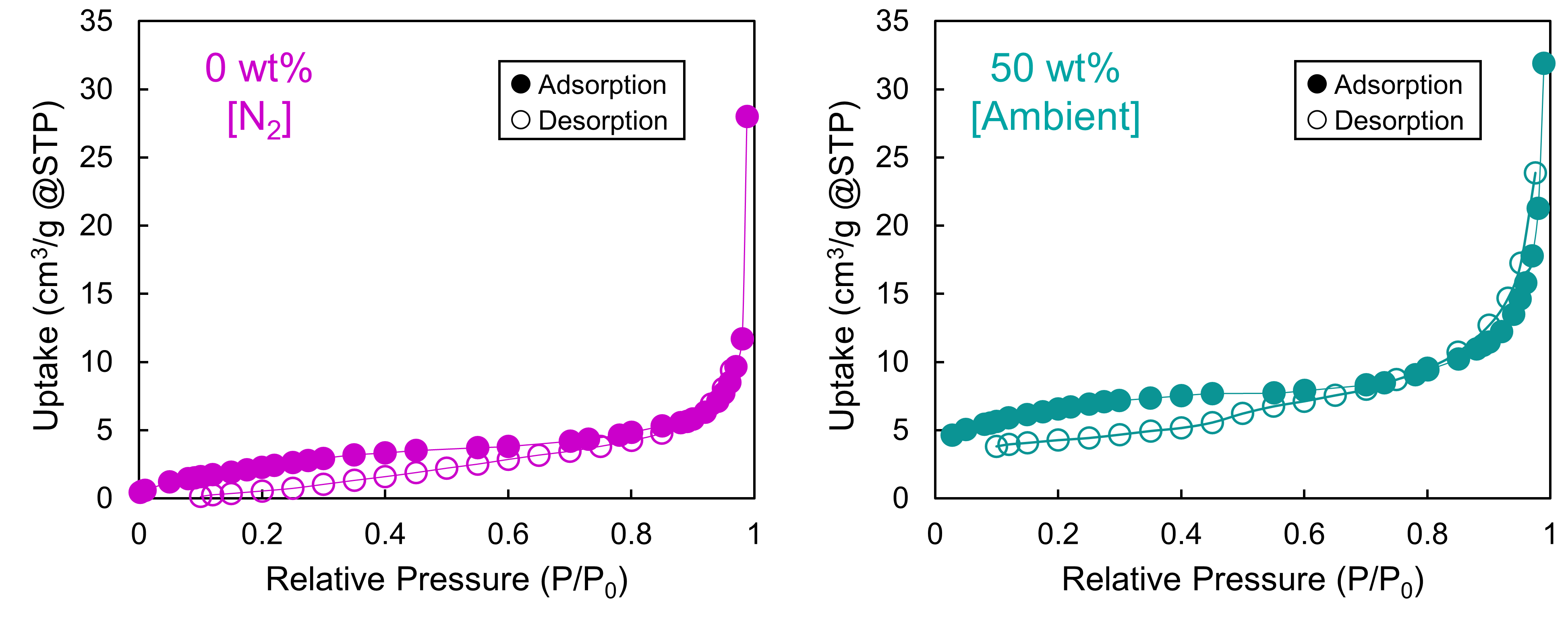


**Figure S26.** Nitrogen adsorption–desorption isotherms of salt-free GAs printed in N_2_ (pink) and salt-assisted GAs printed in ambient conditions (blue). Both samples exhibit comparable N_2_ uptake despite pronounced differences observed by CO_2_ adsorption and electrochemical performance, indicating that conventional N_2_ physisorption is insensitive to the additional ultrafine porosity introduced by salt-assisted laser processing. The isotherms show poorly defined adsorption–desorption hysteresis and non-ideal profiles that do not conform to classical isotherm types.


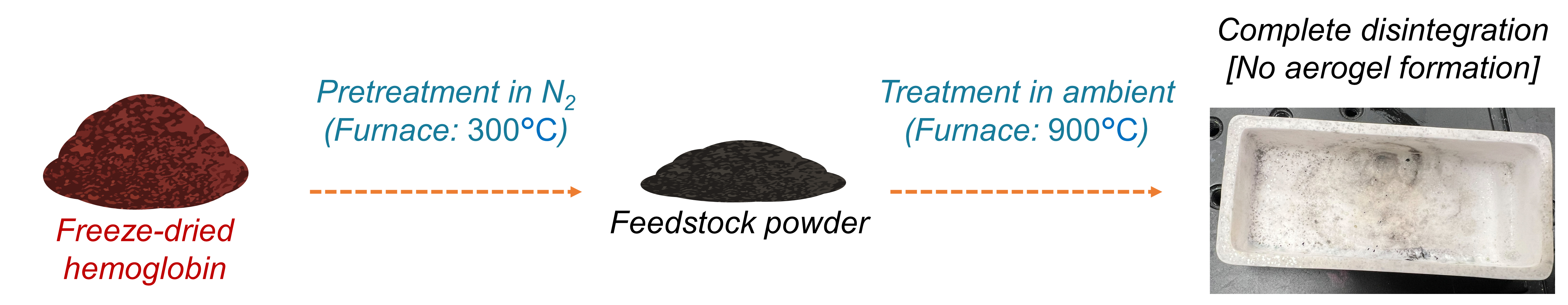


**Figure S27.** Control experiment using furnace as a heat source. Methodology involved the combination of precursor pretreatment adapted in this work, with the methodology presented previously on the fabrication of GAs through pyrolysis of protein-based feedstocks.^1,2^ Following this methodology, no monolithic structures were obtained, due to the complete disintegration of the feedstock powder due to combustion in air.


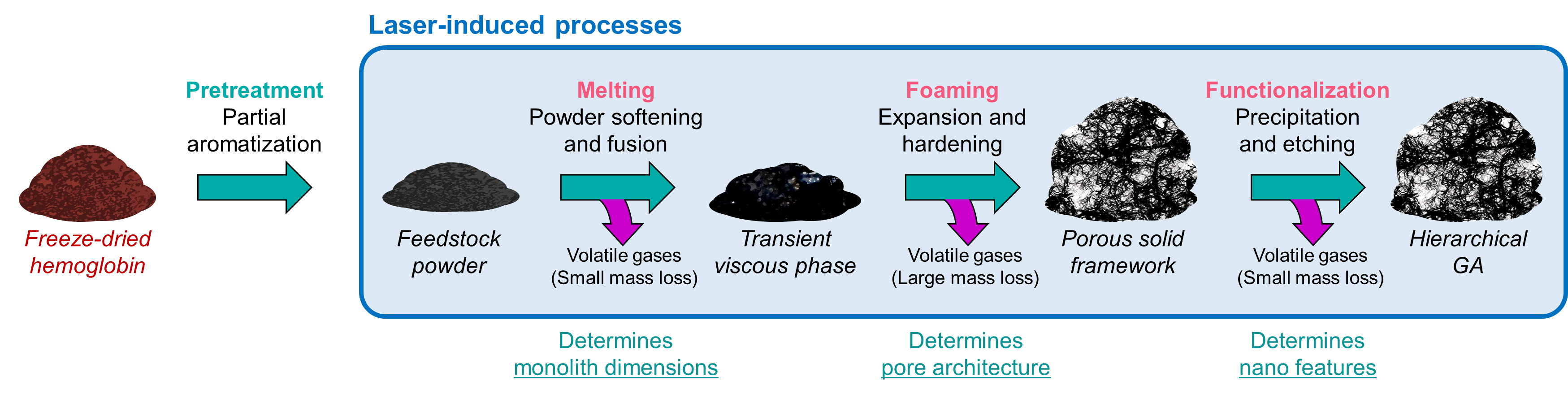


**Figure S28.** Schematic illustration of laser-induced material evolution. Under laser exposure, the feedstock powders soften and coalesce into a transient viscous phase, enabling interparticle fusion into a monolithic structure. Continued pyrolysis drives volatile release, generating bubbles that induce self-foaming while concurrent carbonization solidifies the framework into a porous solid framework. Additional vapor-phase and reactive processes, governed by feedstock composition and environment, further introduce hierarchical nano features as summarized in **Table 1**.


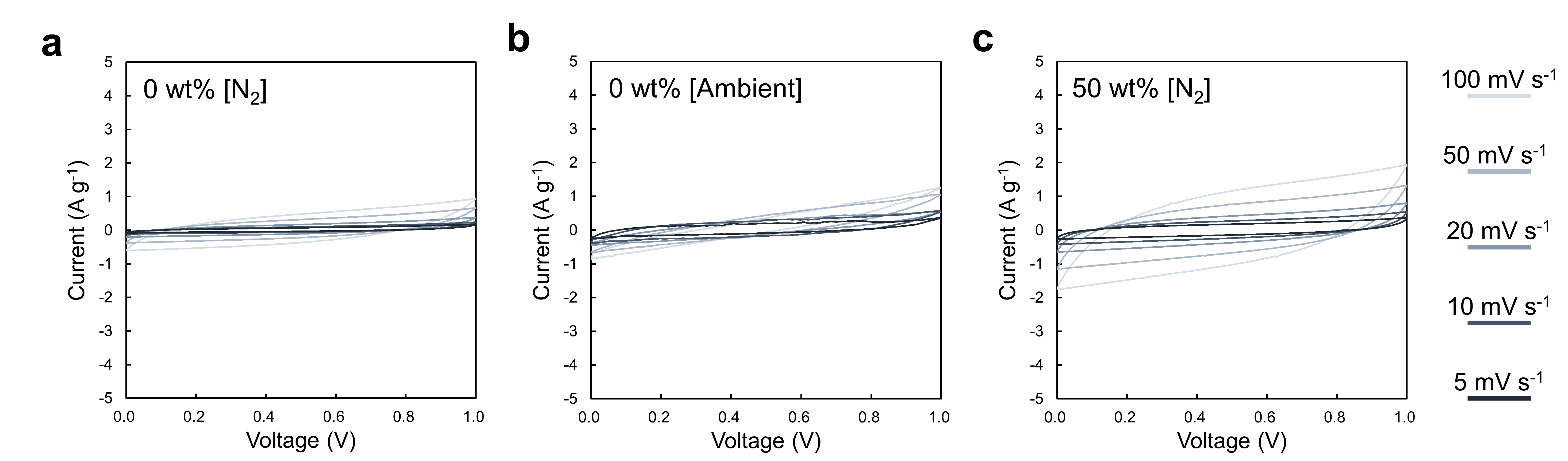


**Figure S29.** CV curves for salt-free GAs printed in **a** N_2_ and **b** ambient, as well as **c** salt-assisted GA printed in N_2_ at scan rates of 100, 50, 20, 10, and 5 mV s^-1^ as color coded.


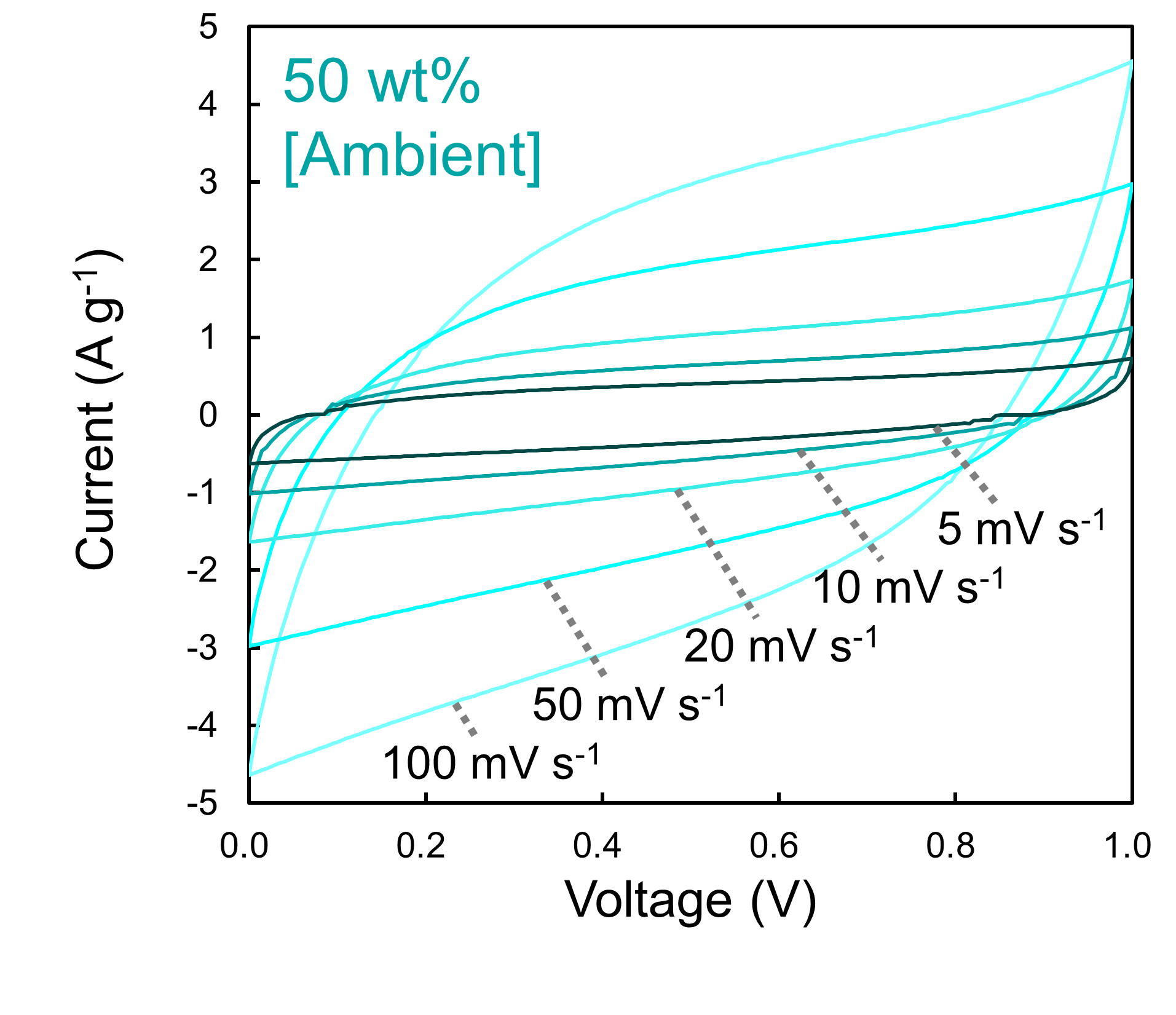


**Figure S30.** CV curves for salt-assisted GAs printed in ambient at different scan rates.


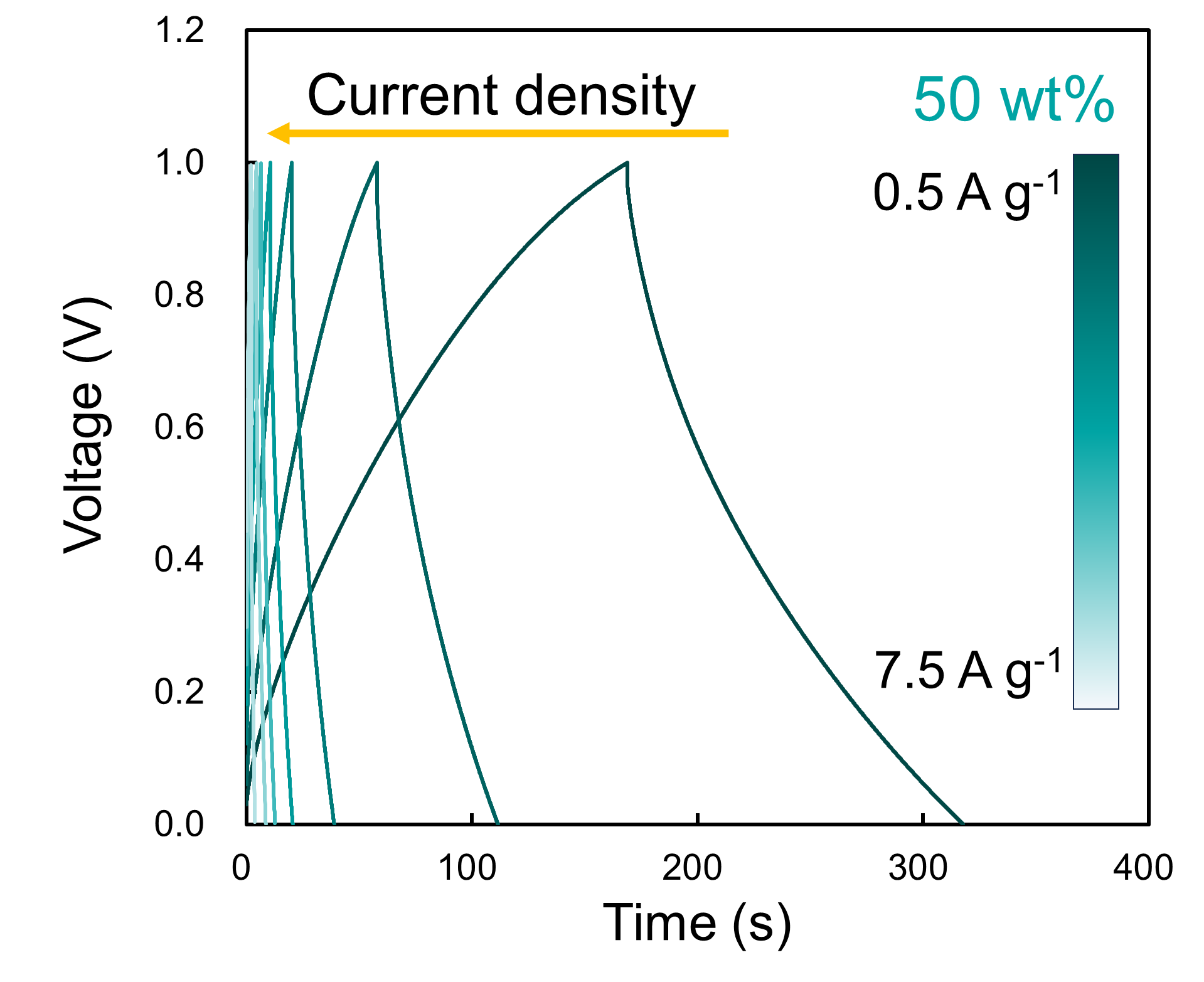


**Figure S31.** CP curves for salt-assisted GAs printed in ambient at various current densities.


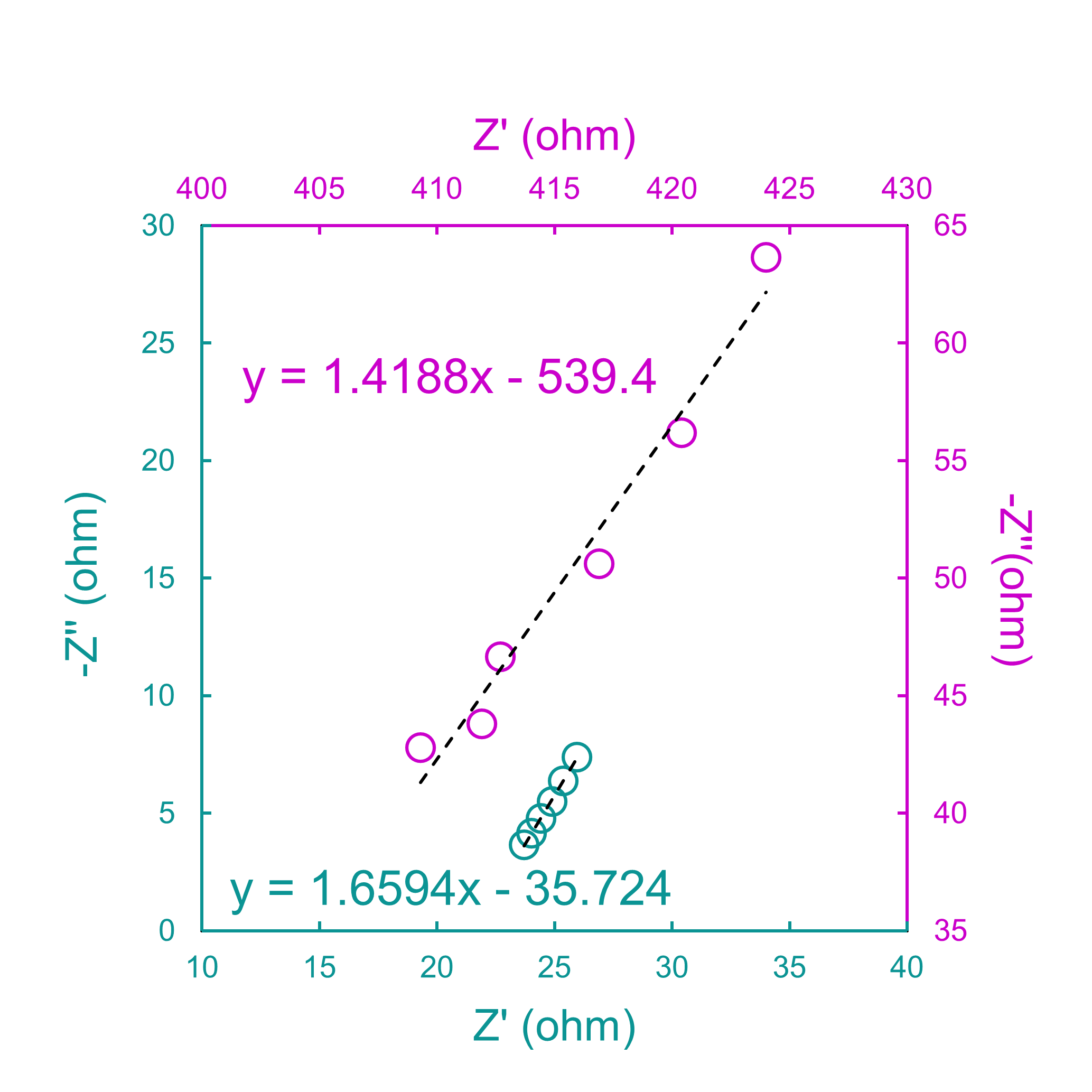


**Figure S32.** Expanded Nyquist plots for the low-frequency region. Linear trend lines reveal the slope of the tail of the Nyquist plot.


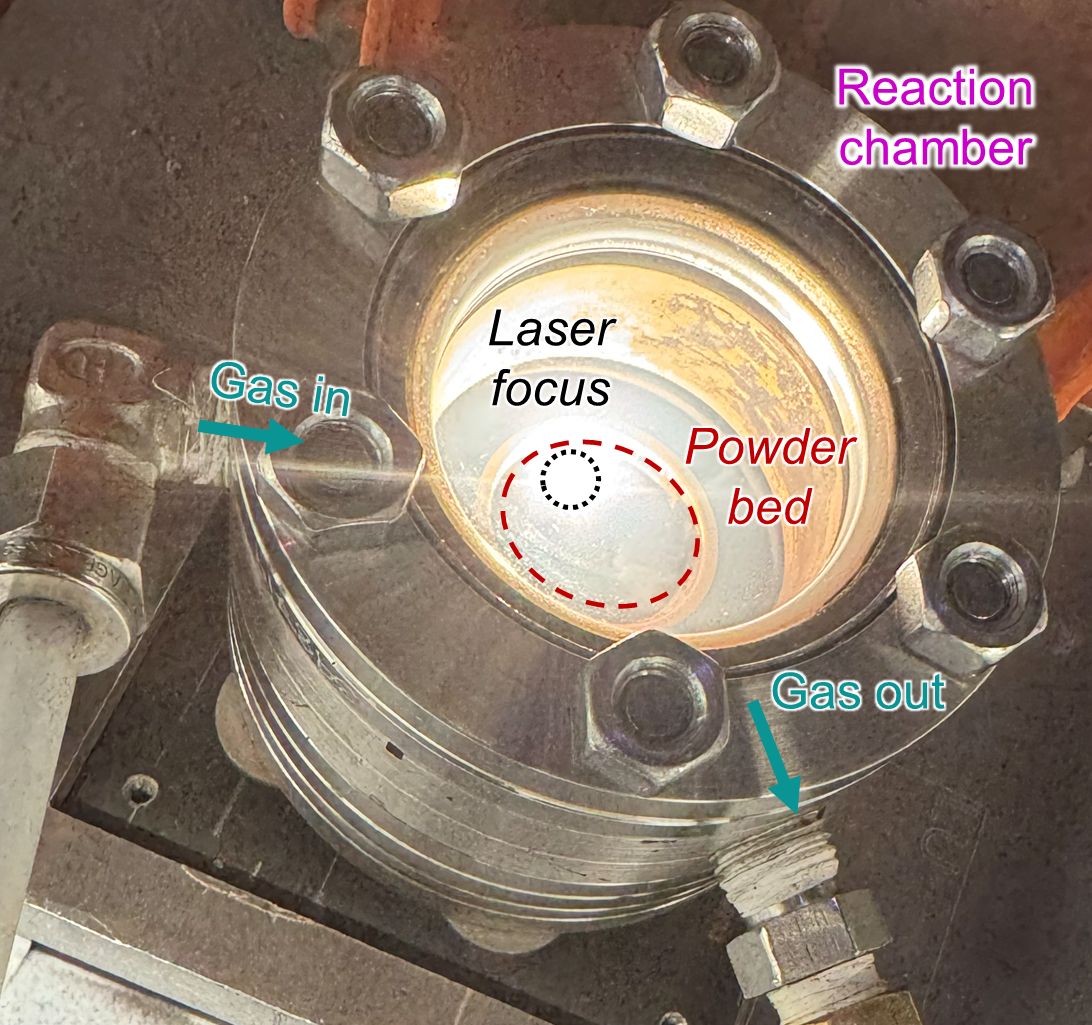


**Figure S33.** Photograph of the laser printing process occurring in the windowed flow chamber equipped with a gas inlet and a vacuum outlet.


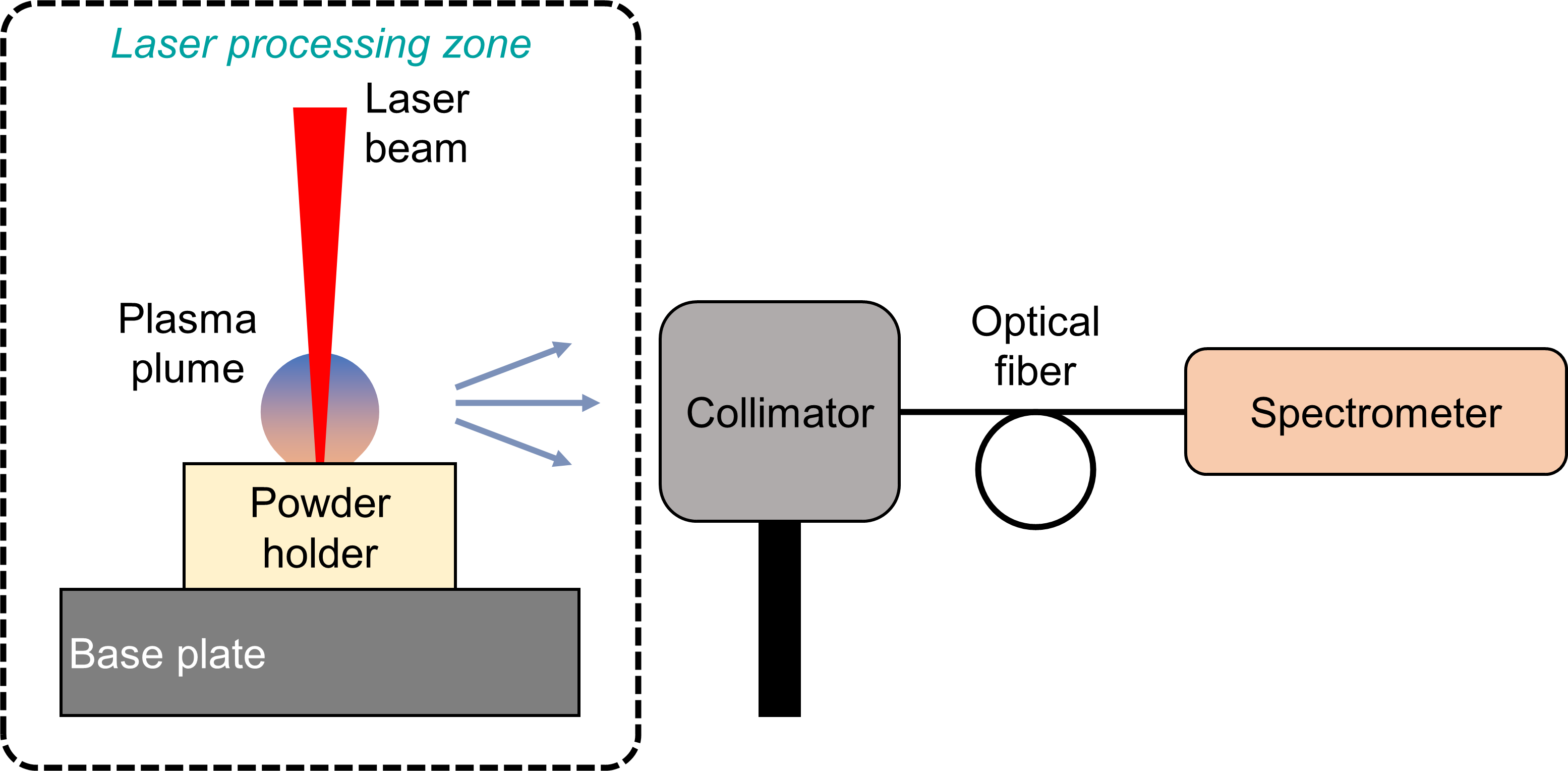


**Figure S34.** Schematic illustration of the setup for optical emission spectra measurements.

**Table S1.** Performance and design metrics used for comparison in the Radar plot.

| Parameter | | This Work | Extrusion Ink  AM [GA] | Hydrothermal  Reduction [GA] | Extrusion Ink AM [AC] | Thin-film  Pyrolysis [AC] |
| --- | --- | --- | --- | --- | --- | --- |
| Gravimetric Capacitance (F g^-1^) | | ~162 | ~186 | ~213 | ~25.6 | <<1 |
| Power Density (W kg⁻¹) | | ~2000 | ~5000 | ~10000 | ~830 | ~3000 |
| Energy Density (Wh kg⁻¹) | | ~6 | ~6.5 | ~20 | ~0.88 | ~0.2 |
| Structural Tunability (Levels) | | 5 | 3 | 2 | 3 | 1 |
| Scalability and Sustainability | Fabrication Time | ~30 s;  near-instant;  minimal pre/post-processing | >>24 h;  multi-step;  low throughput | >>24 h;  batch-capable;  slow kinetics | >>24 h;  multi-step;  limited throughput | >>24 h;  multi-step;  roll-to-roll  compatible |
|  | Material  Sustainability and Flexibility | Broad precursor tunability;  renewable  feedstocks;  minimal waste | Narrow feedstock window;  formulation- limited;  hazardous  chemicals | Broad precursor range;  few constraints;  hazardous  chemicals | Narrow feedstock window;  moderate  sustainability;  fewer hazardous chemicals than GAs | Moderate precursor flexibility;  binders required;  renewable  feedstocks |

**Table S2.** Parameters used in COMSOL simulations for laser-induced temperature profiles.

| Parameter | Variable | Value | Reference |
| --- | --- | --- | --- |
| Laser power [W] | P | 10 |  |
| Scan speed [mm s^-1^] | v | 2.00 |  |
| Absorptivity | A | 0.9 | ^3^ |
| Absorption coefficient [cm^-1^] | A_c_ | 20 | ^4^ |
| Beam radius [μm] | σ_x,_ σ_y_ | 250 |  |
| Porosity | φ | 0.2 |  |
| Weight fraction | ψ | 0.5 |  |
| Thermal conductivity (carbon) [W m^-1^ K^-1^] | k_1_ | 1 | ^5^ |
| Thermal conductivity (salt) [W m^-1^ K^-1^] | k_2_ | 7 | ^6^ |
| Density (carbon) [g cm^3^] | ρ_1_ | 1.5 | ^5^ |
| Density (salt) [g cm^3^] | ρ_2_ | 1.5 | ^6^ |
| Specific heat (carbon) [J g^-1^ K^-1^] | C_1_ | 0.7 |  |
| Specific heat (salt) [J g^-1^ K^-1^] | C_2_ | 0.7 |  |

# **ADDITIONAL DISCUSSIONS**

*VLS-like growth process*

The surface topology was examined as a function of distance from the laser focal spot. **Figure S12a** shows a SEM image of the right side of an aerogel printed by scanning a laser beam (50 wt% salt concentration), with the region of laser scanning indicated by the blue arrow. A diverse range of surface features emerges depending on the distance from the focal region. To better investigate the relationship between salt distribution and the resulting microstructures, an un-washed aerogel was intentionally analyzed here.

A single laser scan across the pre-treated protein feedstock yields millimeter-wide aerogel tracks, as previously reported (~3 mm at current energy density). **Figure S12b** shows higher-magnification SEM images from various distances from the scan path (d), labeled in **Figure S12a**. At location A (d ≈ 0 μm, **Figure S12b_A_**), graphitic microtubes are observed on the aerogel surface. However, the region closest to the focal point (left side of **Figure S12b_A_**) appears relatively smooth and lacks microtube features. This is likely due to elevated peak temperatures near the focal point, which can degrade or ablate the fragile graphitic microtubes, similarly reported for CW-laser processing of carbon aerogels.

At locations B (d ≈ 100 μm, **Figure S12b_B_**) and C (d ≈ 300 μm, **Figure S12b_C_**), compared to location A, longer and more distinct microtubes are observed. Notably, those at location B exhibit closed tips, while those at location C predominantly display open tips. According to TEM observations, this may suggest the formation of thicker-walled microtubes closer to the focal spot. At location D (d ≈ 500 μm, **Figure S12b_D_**), graphitic microspheres with large visible masses begin to appear. At location E (d ≈ 600 μm, **Figure S12b_E_**), thick pillar-like structures emerge. These differ morphologically from microtubes, appearing both wider and denser. At location F (d ≈ 700 μm, **Figure S12b_F_**), smaller dense particles dominate the surface. By location G (d ≈ 900 μm, **Figure S12b_G_**), the surface is covered by a highly compact aggregation of particles, many of which are cubic, indicative of salt crystallites with an average diameter of ~800 ± 200 nm. Finally, at location H (d ≈ 1000 μm, **Figure S12b_H_**), particle density markedly decreases, exposing the underlying aerogel surface.^7^

The variation in elemental composition as a function of distance from the laser focal spot was analyzed using EDS (**Figure S13**). At regions close to the focal spot (d ≤ 300 μm), including locations A, B, and C, the surfaces were predominantly composed of carbon, with minor traces of sodium, chloride, and potassium. Notably, almost no oxygen signal was detected for such regions, suggesting a high degree of pyrolysis with minimal oxidation. These findings are consistent with SEM observations, which revealed dense forests of graphitic microtubes on the surface.

Beyond 300 μm, a sharp decline in carbon content was observed, accompanied by a corresponding rise in sodium, chloride, and potassium concentration. Therefore, the masses and pillar-like features observed at locations D (**Figure S12b_D_**) and E (**Figure S12b_E_**), respectively, are mostly salt-based rather than graphitic structures. At distances of 700–800 μm, including location F, the carbon signal was nearly undetectable, indicating that the top surface was composed almost entirely of salt elements. This indicates that the entire surface consisted of salt elements. However, past this region (d > 800 μm), the carbon concentration increased rapidly, reaching a maximum of ~100% at around d ≈ 1500 μm, corresponding to the edge of the aerogel. This apparent increase in carbon content is attributable to the emergence of the underlying aerogel surface as the salt crystallites decrease in grain size, layer thickness, and overall surface coverage.

The resulting temperature profile as a function of distance from the focal spot is shown in **Figure S14**. At the focal spot, peak temperatures exceeding 1600 °C are predicted, consistent with previous reports on laser pyrolysis and well above the threshold for graphitization.^8^ With increasing distance, the temperature rapidly declines, reaching <400 °C at ~1500 μm. Notably, ~400 °C represents the minimum temperature required for converting protein-based precursors into GAs.^2,9^ The close match between this theoretical threshold, simulated edge temperature, and experimentally observed edge of the printed aerogel suggests that future optimization of the thermal distribution could enable improved printing resolution and control over aerogel width.

Based on the melting (~770 °C) and boiling (~1413 °C) temperatures of the salt additives (NaCl and KCl), the temperature distribution in **Figure S14** can be divided into three distinct regimes. When these thermal regimes are correlated with the observed microstructural features (**Figure S12b**) and elemental compositions (**Figure S13**), a consistent trend emerges. In regions where the estimated temperature exceeds the boiling point of the salt mixture (T > 1413 °C), minimal salt signal is detected by EDS, suggesting that most of the salt has vaporized and dissipated during laser processing. In contrast, regions where the temperature falls between the melting and boiling points of the salts (770 °C < T < 1413 °C) exhibit strong salt signals, indicating that the salts coalesce to form larger molten droplets which become encapsulated by the developing microstructures or condense into larger salt pillars. At temperatures below the melting point of the salts (T < 770 °C), the salt signal again decreases substantially, likely due to the salts remaining in a solid phase, preventing coalescence and growth to completely cover the structure surface.

These observations suggest that while the bulk GA framework forms primarily through laser pyrolysis of the protein precursor, the surface features develop secondarily via a salt-assisted VLS-like mechanism during printing.^10–12^ The classical VLS mechanism for carbon tube growth involves three key components: a solid substrate, a low-melting-point additive (typically a metal catalyst), and a carbon-containing vapor. Upon thermal activation, the additive melts to form liquid droplets on the substrate surface that serve as nucleation sites. When supersaturated with carbon vapor, anisotropic precipitation occurs at the liquid–solid interface initiates tubular growth.

In our system, laser-induced heating establishes a transient multiphase environment analogous to that required for VLS growth (**Figure S15**). Here, the initially formed GA surface acts as the solid substrate, molten alkali halide droplets serve as the liquid template, and carbon-rich vapors released during laser pyrolysis constitute the vapor precursor. Upon irradiation, the protein-based powder rapidly decomposes, releasing carbonaceous vapors while simultaneously forming the solid aerogel framework. Due to low optical absorption in the infrared (**Figure S16**), the salt particles (**Figure S17**) are heated indirectly via conduction from the surrounding matrix, leading to localized melting or vaporization after the powder heats.

Simultaneously, carbon-rich vapors generated at the focal volume preferentially adsorb onto and nucleate upon the molten salt droplets (**Figure S15a**), initiating graphitic layer formation (**Figure S15b**). The eutectic mixture melts at temperatures that overlap with the graphitization regime,^13,14^ thereby facilitating templated growth of graphitic microstructures. At lower salt concentrations, smaller coalesced droplets give rise to nanoscale blisters (**Figure 2b**), whereas larger droplets at higher salt loadings yield microspheres (**Figure 2c**). Continued precipitation at the droplet–aerogel interface drives anisotropic growth, resulting in high-aspect-ratio hollow microtubes (**Figure S15c**). Near the focal volume, elevated temperatures and increased carbon flux promote thicker multilayered walls (**Figure S15d**). As the salt vaporizes (**Figure S15e**), the melt volume decreases, leading to tip tapering and salt entrapment consistent with the nib-like morphologies observed experimentally (**Figure S6**). Thermal simulations validate the temperature conditions required for this mechanism (**Figure S14**), revealing focal spot temperatures exceeding 1600 °C,^8^ well above the salt melting and boiling points as well as the graphitization threshold. The temperature then falls sharply to <400 °C about 1500 μm from the focal center, consistent with the dimensions of the printed aerogel. The strong spatial correlation between evolving morphologies (**Figure S12**), elemental profiles (**Figure S13**), and thermal gradients (**Figure S14**) supports the transient VLS-like process governing the *in situ* microstructural self-assembly during printing

*Cyclic reaction process*

Released salt vapors, particularly potassium species as indicated by spectroscopy (**Figure 3c**), can catalytically react with water, oxygen, and carbon dioxide to initiate surface etching and nanoparticle redeposition (**Equations S1, S2**).^15,16^

|  | KCl+H_2_O→KOH+HCl | (S1) |
| --- | --- | --- |
|  | 2KOH+CO_2_→K_2_CO_3_+2H_2_O | (S2) |

The resulting KOH and K_2_CO_3_ strongly oxidize the carbon-rich aerogel at elevated temperatures, forming metallic potassium and CO (**Equations S3, S4**).^17,18^

|  | 2KOH+2C→2K+2CO+H_2_ | (S3) |
| --- | --- | --- |
|  | K_2_CO_3_+2C→2K+3CO | (S4) |

This oxidation–etching process increases surface area, while the CO vapors can recombine via the reverse Boudouard reaction (**Equation S5**) ^19,20^ to form carbonaceous nanoparticles that redeposit onto the aerogel surface, consistent with the observed particulate features (**Figure 3e**). The presence of alkali metals can further catalyze this reaction.

|  | 2CO→C_particles_+CO_2_ | (S5) |
| --- | --- | --- |

Moreover, intermediate metallic potassium formed during these reactions (**Equations S3, S4**) is continuously reoxidized and rehydroxylated by atmospheric oxygen and water to regenerate KOH (**Equation S6**), which can then be recarbonized according to **Equation S2**, closing a catalytic cycle that sustains surface etching and particle formation.^21^

|  | 4K+O_2_+2H_2_O→4KOH | (S6) |
| --- | --- | --- |

In contrast, under inert N_2_ conditions, even with salt addition, these reactions are self-limited by the heteroatom content of the protein-based precursor, resulting in a more controlled and static structural evolution.

# **ADDITIONAL REFERENCES**

1. Ozden, S. *et al.* Egg Protein Derived Ultralightweight Hybrid Monolithic Aerogel For Water Purification. *Materials Today* **59**, 46–55 (2022).

2. Hayashi, S., Das, A., Rupp, M., Wani, M. S. & Arnold, C. B. Freeform monolithic graphitic aerogels by laser pyrolysis of pretreated blood-derived feedstocks. *Matter* **8**, 102432 (2025).

3. Goldberg, P. M. *Proceedings of the Advanced Research and Technology Development Direct Utilization, Instrumentation and Diagnostics Contractors’ Review Meeting*. (Pittsburgh Energy Technology Center, Pittsburgh, PA, USA, 1990).

4. Russo, C., Apicella, B., Lighty, J. S., Ciajolo, A. & Tregrossi, A. Optical properties of organic carbon and soot produced in an inverse diffusion flame. *Carbon N. Y.* **124**, 372–379 (2017).

5. Wang, Y., Fan, Z., Qian, P., Caro, M. A. & Ala-Nissila, T. Density dependence of thermal conductivity in nanoporous and amorphous carbon with machine-learned molecular dynamics. *Phys. Rev. B* **111**, (2025).

6. Galamba, N., De Castro, C. A. N. & Ely, J. F. Thermal conductivity of molten alkali halides from equilibrium molecular dynamics simulations. *Journal of Chemical Physics* **120**, 8676–8682 (2004).

7. Hayashi, S., Sanchirico, A., Das, A. & Arnold, C. B. Three-dimensional carbon fiber networks with self-orienting nano-textures enabled by femtosecond laser processing. *Appl. Phys. Lett.* **126**, 041601 (2025).

8. Le, T. S. D. *et al.* Recent Advances in Laser-Induced Graphene: Mechanism, Fabrication, Properties, and Applications in Flexible Electronics. *Adv. Funct. Mater.* **32**, 2205158 (2022).

9. Wani, M. S., Denzer, B., Caggiano, N. J., Prud’homme, R. K. & Arnold, C. B. Hierarchically Porous Graphitic Aerogels via Thermal Morphogenesis of Proteins for Environmental Remediation. *ACS Appl. Nano Mater.* **8**, (2025).

10. Wagner, R. S. & Ellis, W. C. Vapor-liquid-solid mechanism of single crystal growth. *Appl. Phys. Lett.* **4**, 89–90 (1964).

11. Xu, X., Yang, C., Yang, Z., Yang, K. & Huang, S. Carbon nanotube growth from alkali metal salt nanoparticles. *Carbon N. Y.* **80**, 490–495 (2014).

12. Bhimarasetti, G., Cowley, J. M. & Sunkara, M. K. Carbon microtubes: Tuning internal diameters and conical angles. *Nanotechnology* **16**, (2005).

13. Liu, X., Fechler, N. & Antonietti, M. Salt melt synthesis of ceramics, semiconductors and carbon nanostructures. *Chem. Soc. Rev.* **42**, 8237–8265 (2013).

14. Fechler, N., Fellinger, T. P. & Antonietti, M. ‘Salt templating’: A simple and sustainable pathway toward highly porous functional carbons from ionic liquids. *Advanced Materials* **25**, 75–79 (2013).

15. Blomberg, T., Tripathi, T. & Karppinen, M. New chemical mechanism explaining the breakdown of protective oxides on high temperature steels in biomass combustion and gasification plants. *RSC Adv.* **9**, 10034–10048 (2019).

16. Pak, J., Han, S. J. & Wee, J. H. Precipitation of potassium-based carbonates for carbon dioxide fixation via the carbonation and re-carbonation of KOH dissolved aqueous ethanol solutions. *Chemical Engineering Journal* **427**, (2022).

17. Wang, J. & Kaskel, S. KOH activation of carbon-based materials for energy storage. *J. Mater. Chem.* **22**, 23710–23725 (2012).

18. Chun, S. E. & Whitacre, J. F. Formation of micro/mesopores during chemical activation in tailor-made nongraphitic carbons. *Microporous and Mesoporous Materials* **251**, 34–41 (2017).

19. Gómez, I. C., Cruz, O. F., Silvestre-Albero, J., Rambo, C. R. & Escandell, M. M. Role of KCl in activation mechanisms of KOH-chemically activated high surface area carbons. *Journal of CO2 Utilization* **66**, (2022).

20. Kogler, M. *et al.* High-Temperature Carbon Deposition on Oxide Surfaces by CO Disproportionation. *Journal of Physical Chemistry C* **120**, 1795–1807 (2016).

21. Rao, Y. K., Adjorlolo, A. & Haberman, J. H. On the mechanism of catalysis of the Boudouard reaction by Alkali-metal compounds. *Carbon N. Y.* **20**, 207–212 (1982).
